# Advanced Light Source Technologies for Photodynamic Therapy of Skin Cancer Lesions

**DOI:** 10.3390/pharmaceutics15082075

**Published:** 2023-08-03

**Authors:** José Francisco Algorri, José Miguel López-Higuera, Luís Rodríguez-Cobo, Adolfo Cobo

**Affiliations:** 1 Photonics Engineering Group, University of Cantabria, 39005 Santander, Spain; adolfo.cobo@unican.es; 2 CIBER de Bioingeniería, Biomateriales y Nanomedicina, Instituto de Salud Carlos III, 28029 Madrid, Spain; luis.rodriguez@unican.es; 3 Instituto de Investigación Sanitaria Valdecilla (IDIVAL), 39011 Santander, Spain

**Keywords:** photodynamic therapy, skin cancer, reactive oxygen species (ROS), wearable medicine, light-emitting materials, textile diffusers, adhesive devices

## Abstract

Photodynamic therapy (PDT) is an increasingly popular dermatological treatment not only used for life-threatening skin conditions and other tumors but also for cosmetic purposes. PDT has negligible effects on underlying functional structures, enabling tissue regeneration feasibility. PDT uses a photosensitizer (PS) and visible light to create cytotoxic reactive oxygen species, which can damage cellular organelles and trigger cell death. The foundations of modern photodynamic therapy began in the late 19th and early 20th centuries, and in recent times, it has gained more attention due to the development of new sources and PSs. This review focuses on the latest advancements in light technology for PDT in treating skin cancer lesions. It discusses recent research and developments in light-emitting technologies, their potential benefits and drawbacks, and their implications for clinical practice. Finally, this review summarizes key findings and discusses their implications for the use of PDT in skin cancer treatment, highlighting the limitations of current approaches and providing insights into future research directions to improve both the efficacy and safety of PDT. This review aims to provide a comprehensive understanding of PDT for skin cancer treatment, covering various aspects ranging from the underlying mechanisms to the latest technological advancements in the field.

## 1. Introduction

Skin cancer starts in the skin and is often (but not always) caused by extra-exposure to ultraviolet (UV) radiation from the sun or artificial sources, such as tanning beds. Skin cancer is the most common type globally, with an estimated 5.4 million cases diagnosed yearly in the United States alone (occurring in about 3.3 million Americans, as some people are diagnosed more than once) [1]. In terms of melanoma, the crude global incidence in Spain is about 8.82 cases per 100,000 people/year, and the crude global mortality is 2.17 cases per 100,000 people/year. This rate is relatively lower when compared to several other countries in Europe, as well as the USA, Australia, and New Zealand [2]. For squamous cell carcinoma (SCC), a meta-analysis from 2016 determined the crude incidence rate in Spain to be 38.16 per 100,000 person-years [3]. This puts Spain on the lower end of incidence rates when compared with countries like Germany and Slovakia. Several factors may influence these rates, including geography, sun exposure habits, socioeconomic conditions, and smoking patterns. The analysis in reference [2] shows a trend of increased mortality in the eastern provinces of Spain, potentially due to a greater beach culture and the associated intermittent sun exposure.

While early detection can lead to a cure, some forms of skin cancer can be aggressive and hard to treat. Photodynamic therapy (PDT), initially developed in the 20th century [4] (the first literature reports of a “photodynamic effect” were provided by Raab and von Tappeiner [5]), has seen a significant resurgence in recent years as an effective, non-invasive treatment for skin cancer. This therapeutic approach, owing to its selectivity and efficiency, has garnered considerable attention in the medical field [6]. The mechanism of PDT revolves around the utilization of a photosensitizing agent, a special kind of molecule that can be activated by specific wavelengths of light. Once the photosensitizing agent has been administered, usually through an injection or a topical cream, it accumulates in the cancerous cells over a period of time. When the affected area is exposed to a specific type of light, the photosensitizing agent becomes excited, resulting in the generation of reactive oxygen species (ROS) [7], including the highly reactive singlet oxygen. The principle of PDT is to selectively destroy the abnormal and cancerous cells, leaving the healthy ones and cytoskeleton unscathed, which is what makes the repair process easy without too much scarring, leading to good cosmetic results compared to cryotherapy or surgery. The key to this selectivity lies in the action of the light-activated photosensitizing agents. When the light excites these agents, they transition to an energized state. This excited state then interacts with molecular oxygen, which leads to the production of singlet oxygen, a highly reactive molecular species [8]. Singlet oxygen is a critical player in the mechanism of PDT. It is produced through the Type II process in PDT, a term referring to the energy transfer between the excited photosensitizing agent and molecular oxygen. This leads to the generation of singlet oxygen, which has a higher energy state than regular molecular oxygen. Its enhanced reactivity makes it a potent weapon against the abnormal cells, causing significant cytotoxic effects [9]. These cytotoxic effects, essentially, are the root cause of the cellular damage seen in the targeted cancerous cells. The highly reactive singlet oxygen can disrupt cellular processes, damage the cellular structures, and even induce programmed cell death, also known as apoptosis (also, necrosis and autophagy can be produced) [10]. Thus, the singlet oxygen, through its potent cytotoxic effects, plays a pivotal role in the efficacy of PDT [11]. In the next section, we will delve deeper into the mechanism of singlet oxygen production and its role in PDT. This includes exploring how the energy transfer occurs during the Type II process, how the singlet oxygen interacts with the cellular components to induce cytotoxic effects, and what factors may affect the efficiency of singlet oxygen production.

The use of PDT in treating skin cancer stands out as particularly significant for several reasons. Firstly, skin cancers are typically located at or near the skin surface, making them readily accessible to both the application of the photosensitizer and the light necessary for PDT. Secondly, skin cancers often occur in cosmetically sensitive areas where tissue-sparing treatments like PDT can preserve appearance and function. Furthermore, unlike internal cancers, the treatment response of skin cancers can be visually monitored, making it easier to assess the effectiveness of PDT. Lastly, the non-invasive nature of PDT coupled with its negligible effects on underlying functional structures make it an ideal treatment modality for skin cancer, allowing for feasible tissue regeneration. Most skin lesions are treated by topically applying photosensitizers (PSs) like aminolevulinic acid (ALA) or methyl aminolevulinate (MAL) [12,13,14]. However, this topical approach allows only 1–2 mm of light penetration into the tissue, which is insufficient for treating deep lesions [15]. Moreover, PDT has been shown to be effective in treating various types of skin cancer, including basal cell carcinoma (BCC), squamous cell carcinoma (SCC), and actinic keratosis (AK). AK is a pre-cancerous skin lesion that can progress to SCC if left untreated (which can, in turn, metastasize if left untreated). The use of photosensitizing agents like 5-aminolevulinic acid or methyl aminolevulinate (MAL) in conventional PDT has proven to be an effective treatment for AK, especially when dealing with extensive areas of field change, as indicated by several studies [16,17,18]. Even with daylight, PDT provides better outcomes (see Section 5.1.1). The main findings of study [19] suggest that for Bowen’s disease (BD), PDT may lead to better outcomes compared to cryotherapy or fluorouracil. Reference [20] supports the use of topical 5-Fluorouracil (5-FU) PDT as an effective therapy for BD, with cosmetic outcomes superior to standard treatment. BCC is the most common form of skin cancer, accounting for 80% of cases [1]. PDT is approved in Europe for treating superficial basal cell tumors of the skin, with a reported 95% response rate among patients who had a complete response to therapy [15,21]. PDT of MAL may also provide similar lesion response rates to surgery or cryotherapy for BCC [20], but the efficacy may be lower in nodular BCC, making surgery a better option in some cases. A systematic review and meta-analysis in [22] showed that while PDT of MAL may not be the best first-line treatment for BCC, it can sometimes provide excellent cosmetic outcomes. The use of Temoporfin (mTHPC)-PDT for non-melanoma skin cancer (NMSK) is promising, but there is insufficient evidence to fully assess its efficacy as a first-line treatment [23]. Moreover, it has to be taken into account that surgery is the recommended treatment for this rapidly spreading cancer. For SCC, PDT is recommended for treating in situ lesions. Research shows that after 1–2 cycles of PDT of MAL, lesion clearance rates range from 88 to 100%, with no recurrence in 68–89% of treated lesions at a follow-up of 17–50 months (as reported in studies [24,25,26,27,28]). However, there is currently limited evidence supporting the effectiveness of PDT for invasive SCC. The primary reasons for the limitations of topical PDT may be inadequate penetration of the photosensitizer applied topically through tumor tissue or reduced cellular uptake due to insufficient local bioavailability [6]. To solve these problems, researchers have proposed different alternatives, such as intralesional injection of PS [20] or nanomedicine to encapsulate the PS [29,30].

PDT can be used alone or in combination with other treatments, such as topical therapies or systemic medications, to improve treatment outcomes. A combined strategy of PDT with other modalities, such as immunomodulatory agents, chemotherapeutic agents, inhibitors of carcinogenic molecules, surgical techniques, or radiotherapy, can be effective depending on the tumor type and characteristics [31]. Other research is focused on improving the efficacy of PDT, reducing side effects, and expanding its clinical applications by developing new PSs that are more selective for cancer cells and have a higher activation efficiency (third generation). Another area of research is the optimization of treatment parameters, such as light dose, light intensity, and PS dose, to maximize treatment efficacy while minimizing side effects. Despite the good efficacy and tolerability of PDT in clinical trials, it is not currently a first-line treatment option. However, with continued advancements in technology and research, PDT has the potential to become a standard of care in the future, particularly for superficial BCC and AK. It is important for clinicians and researchers to continue exploring this promising treatment option and developing new strategies to optimize its use in the treatment of skin cancer.

In summary, PDT is a promising non-invasive treatment option for skin cancer that has shown good efficacy and tolerability in clinical trials. Ongoing research is focused on improving its efficacy, reducing side effects, and expanding its clinical applications. With continued advancements in technology and research, PDT has the potential to revolutionize skin cancer, and a combination of PDT with other therapies, such as immunotherapy and chemotherapy, may offer better outcomes than PDT alone. By combining different treatments, it may be possible to target different aspects of the cancer cells and improve the overall effectiveness of the treatment. Ongoing research in this area is crucial for developing better treatment options for skin cancer patients and improving their quality of life. Recent reviews have focused on different aspects, such as the combination of PDT with other treatments [31], 5-ALA- [22] and mTHPC-mediated [23] PDT, use of light-emitting diodes (LEDs) [32], lasers [33], daylight [34,35,36], and fabrics [37,38,39], general updates [40,41], and immune consequences [42], among others topics (see Ref. [43] for a complete compendium of the latest reviews). Some of the previous reviews have covered the use of light sources, as we include in Section 4. But none have focused on advanced light sources and delivery devices. For this reason, our review covers the most recent developments in light source technologies and delivery devices utilized for treating skin cancer, which is a subject that has not been covered explicitly in any previous review. This review follows a well-structured format to provide a comprehensive understanding of advanced light sources for treating skin cancer. Firstly, this review starts with a concise introduction to the underlying mechanisms of PDT, including the principles of light interaction with PS in the treatment. This section aims to give a fundamental understanding of the PDT process and its importance in treating skin cancer. The characteristics of light sources used in PDT are profoundly influenced by the underlying photodynamic reaction mechanisms (as detailed in Section 2) and the specific requirements of the employed photosensitizers, discussed in Section 3. The latter section delves into the approved PSs, with a particular focus on those approved for the treatment of skin cancer. The section includes a detailed discussion of the characteristics and properties of each PS, highlighting their strengths and weaknesses. Additionally, this review provides insights into the potential risks and side effects associated with using these PSs, helping the readers make informed decisions. The next section focuses on the available techniques and light sources for PDT. Understanding the specific requirements of different PSs for light sources is therefore pivotal for the effective design and application of PDT protocols. It discusses the different techniques and types of light sources used in PDT, including their pros and cons, and the technical specifications required for optimal performance. This section also highlights the challenges associated with light sources and their impact on the effectiveness of PDT for skin cancer treatment. Furthermore, this review dedicates a section to discussing the latest light technologies and advanced illumination techniques. It covers recent research and developments in the field of light-emitting technologies, their potential benefits and drawbacks, and their implications for clinical practice. Lastly, this review concludes by summarizing the key findings and discussing their implications for the use of PDT in the treatment of skin cancer. It highlights the limitations of the current approaches and provides insights into future research directions to improve the efficacy and safety of PDT for skin cancer. Overall, this review aims to provide a comprehensive understanding of PDT for skin cancer treatment, covering various aspects ranging from the underlying mechanisms to the latest technological advancements in the field.

## 2. Mechanism of action

The foundational pillar upon which PDT stands is the highly selective destruction of targeted cells. This mechanism of action is reliant on the use of photosensitizing agents and light working synergistically to achieve the desired effect. Photosensitizers (PSs) are a unique class of molecules with the distinctive ability to absorb light energy and transmute it into a form of chemical energy. This energy is subsequently used to trigger a series of photochemical reactions that ultimately lead to a therapeutic effect. The delivery of these photosensitizing agents is typically topical, with the PS being applied directly to the skin. The cancerous cells absorb the PS, and it is distributed among the different sub-cellular compartments of these cells. The most common localizations are the mitochondria and the endoplasmic reticulum, among other intracellular locations (see next section).

### 2.1. Mechanism of Physical Reaction

**Figure 1:**
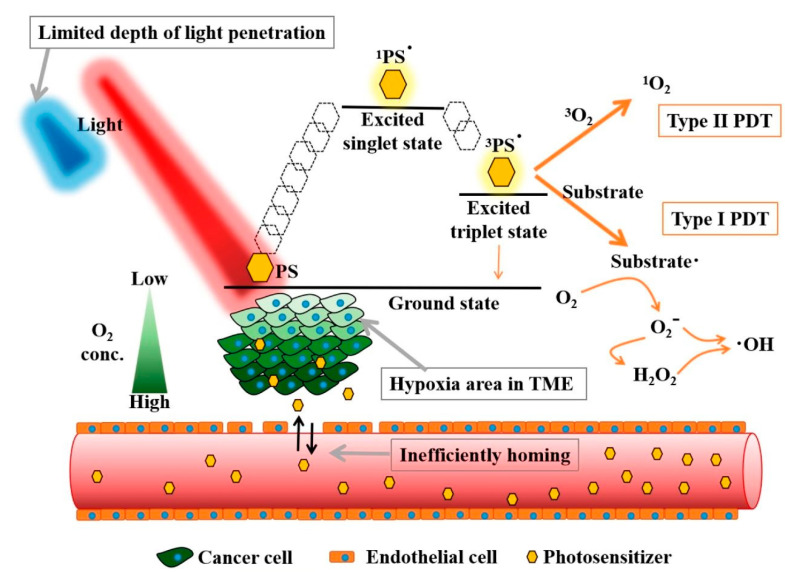
The principle of photodynamic reaction and its basic limitations applied in deep-seated tumor treatment. PS: photosensitizer, TME: tumor microenvironment. Reprinted with permission from Ref. [44]. Copyright year 2021 by the authors. Licensee MDPI, Basel, Switzerland.

Upon the administration of light, the PS becomes excited. As Figure 1 illustrates, this activation through light energy propels the PS to interact with surrounding oxygen molecules. This interaction produces either free radicals or singlet oxygen through the Type I and Type II processes, respectively. The Type II process that results in the generation of singlet oxygen is the most common product of PDT; thus, it forms the core of this discussion. Other photochemical and photophysical processes are undoubtedly part of the PDT process, but our focus rests primarily on those contributing to the simplified explanation of singlet oxygen (^1^PS^•^) production in PDT [44]. The generation of ^1^PS^•^ by a photosensitizer is initiated through a process similar to fluorescence. Upon absorption of a photon with an appropriate wavelength, the photosensitizer molecule transitions into an excited electronic state (Figure 1).

In conventional fluore scence, this excited state experiences a minor non-radiative de-excitation within its surroundings, followed by the emission of a photon at a lower (red-shifted) energy level. However, photosensitizers can undergo de-excitation via an alternative pathway, where the excited-state molecule undergoes intersystem crossing (ISC) to reach a triplet state (^3^PS^•^). While in the ^3^PS^•^, the PS has the ability to transfer energy to nearby oxygen molecules. This transfer is critical to the PDT mechanism as it leads to the formation of singlet oxygen (^1^O_2_). Additionally, it causes the PS to return to the ground state. Singlet oxygen is highly reactive and it interacts with the surrounding cells, leading to cytotoxic effects, which are central to the cell-killing capability of PDT. Moreover, the same PS molecule has the potential to generate multiple singlet oxygen molecules, amplifying the destructive effect. It is worth noting that the ^3^PS^•^ requires an additional energy amount beyond the 0.974 eV direct energy transfer for the irreversible formation of singlet oxygen. This sets the minimum energy requirement for the process at 1.13 eV, which is comfortably met by most PSs [45]. While the focus here has been primarily on the generation of singlet oxygen and the basic mechanisms of PDT, it is important to note that there are other processes and energy transitions that can influence the PDT mechanism. Readers interested in a deeper exploration of these competing processes are encouraged to consult the detailed discussions in references [46,47]. The in-depth insights provided in these sources offer a greater understanding of the intricate mechanisms underlying the field of PDT.

To maximize the therapeutic effect of PDT, the photosensitizer should be able to produce ROS at a level sufficient to induce cancer cell death while minimizing damage to normal cells. Several factors can influence the efficiency of ROS production by a photosensitizer, including:

- Absorption efficiency: The photosensitizer should be able to absorb light efficiently at a wavelength that can penetrate the skin to reach the target cells. The absorption spectrum of the photosensitizer should be well-matched to the wavelength of the light source used for activation to ensure maximum energy transfer and ROS generation.
- Quantum yield: The quantum yield of a photosensitizer refers to the ratio of the number of singlet oxygen molecules generated to the total number of absorbed photons. Higher quantum yields indicate a greater efficiency in generating singlet oxygen, potentially leading to increased ROS production and a more effective therapeutic response.
- Oxygen availability: ROS production by the photosensitizer requires the presence of molecular oxygen, which is sometimes limited in tumor tissues (see Figure 1). Photosensitizers that can generate ROS under low-oxygen conditions are more effective in PDT.
- Fluence rate of light illumination (W/cm^2^): Molecular oxygen in the tissue to be treated is critical for photodynamic cell killing. Oxygen depletion can appear at high fluence rates, diminishing or even totally inhibiting tumor-killing effectiveness during the PDT process.

### 2.2. Mechanism of Biological Events

In addition to the production of ROS, the activation of the PS also triggers a cascade of other biological events that contribute to the efficacy of PDT. One of these events is the release of cytokines and chemokines that recruit immune cells to the site of the treated lesion. This immune response can help to clear the remaining cancer cells and prevent the recurrence of the lesion [48]. Additionally, PDT can induce apoptosis, a form of programmed cell death, in cancer cells. This process involves the activation of signaling pathways that lead to the fragmentation of DNA and the degradation of cellular components, ultimately resulting in the death of the cancer cell. Reference details on cell death pathways can be found in [9,10,11,49,50,51,52]. Another important aspect of the mechanism of action of PDT is the concept of the “bystander effect” [53]. This refers to the ability of PDT to induce damage not only in the cells that directly absorb the PS but also in neighboring cells that have not been directly exposed to the PS. This occurs through the diffusion of the ROS from the treated cells to the surrounding tissue, causing damage to adjacent cells. The bystander effect can increase the overall efficacy of PDT by targeting a larger area of the tumor and reducing the likelihood of disease recurrence. The efficacy of PDT is also influenced by various factors, such as the type of PS used, the dose of PS and light, the time interval between PS administration and light exposure, and the wavelength and intensity of the light used to activate the PS. The choice of PS is critical for PDT success, and several factors should be considered, such as its absorption spectrum, pharmacokinetics, and toxicity profile. The dose of PS and light must be carefully optimized to achieve the desired level of treatment efficacy while minimizing side effects.

Overall, without considering its indirect effect (activation of the immune system), an optimized PDT process engineering must be implemented to reach an optimum result of the cancer cells’ killing. It can be obtained by optimizing the overlap integral of the spatial volume distributions in the diseased tissue, its inside molecular oxygen distribution, its inside photosensitizer distribution, and the illuminating light volumetric distribution inside the cancer volume. The higher the overlap integral, the greater the amount of diseased tissue that is killed, as well as the specificity (no ROS in healthy tissue), resulting in higher effectiveness and efficiency of the photodynamic process.

The complex mechanism of action of PDT makes it a highly selective and effective treatment option for skin cancer. Its ability to target specific areas of the skin, damage nearby cancer cells, induce an immune response, and be repeated as necessary without causing cumulative toxicity or harming healthy tissue makes it a promising option for the treatment of various types of skin cancer. The efficacy of PDT is influenced by various factors, such as the type of PS used, the dose of PS and light, the time interval between PS administration and light exposure, and the wavelength and intensity of the light used to activate the PS. Oxygen depletion (diminishing tumor-killing effectiveness) as a consequence of continuous insolation light with high fluence rates must also be avoided by appropriately modulating the light beam [54]. Therefore, the modulating signal pattern of the amplitude-modulated beam must be designed to recover the potential oxygen depletions (along the maximum intensity time) during the lower-intensity period.

Due to the need for precise control over multiple factors, obtaining reproducible outcomes in PDT is challenging. This variability has contributed to the limited adoption of PDT in clinical settings. Nevertheless, with ongoing research and technological advancements, PDT holds promise as a valuable tool in combating skin cancer.

## 3. Approved PSs for Skin Cancer Diseases

While there are many potential PSs for PDT, only a few have been approved for use in the treatment of skin cancer or other conditions. This is partly due to the rigorous testing and regulatory approval process that new drugs and therapies must undergo before being marketed to the public. In addition, developing effective PSs for PDT is a complex process that involves balancing a number of factors, including: the ability to selectively target cancer cells, the efficiency of ROS production, and the ability to be activated by light in a specific wavelength range. Many potential PSs may not meet all of these criteria or may have other limitations that prevent them from being suitable for clinical use.

Most commonly used PSs can be classified into three generations based on their structures and properties. The first-generation PSs used in PDT were developed in the 1970s and were based on Hematoporphyrin Derivative (HpD), a mixture of porphyrins extracted from a crude preparation of hematoporphyrin. HpD and subsequent derivatives were used in clinical practice for several decades and showed promising results in the treatment of various cancers. However, these first-generation PSs, including Photofrin, introduced later, had several limitations that restricted their use [55]. These PSs are known to exhibit long-lasting photosensitizing effects on the skin, which can lead to photosensitivity reactions and skin damage. However, it should be noted that the production of ROS in normal tissues by first-generation PSs is not primarily attributed to their broad absorption spectrum. The second-generation PSs, on the other hand, are either single agents used alone or in combination, and they show improved absorption within the optimal therapeutic window. These PSs include chlorins, bacteriochlorins, and phthalocyanines. They have better photophysical properties, such as higher quantum yields, and lower dark toxicity. These PSs have been extensively investigated and are widely used in PDT.

Finally, third-generation PSs are designed using certain strategies, such as bioconjugation or encapsulation, to increase specificity in targeting pathological tissue [56]. In ideal cases, the target molecule should be commonly yet selectively expressed by multiple tumor compartments, such as cancer cells, tumor vascular endothelial cells, cancer stem cells, and myeloid-derived suppressor cells [57]. Another approach involves using nanotechnology to create nanocapsules or nanospheres with diameters below 100 nm that can act as drug carriers. These drug carriers offer several advantages, such as being able to transport hydrophobic drugs in blood, having large distribution volumes, and having controlled release capabilities [58]. With the promise of total targeted therapy, the third-generation PSs offer selective attachment to specific tumor cells, reducing toxicity to normal cells and limiting off-target effects [59]. Classifying PSs into three generations provides a useful framework to understand their properties and potential applications in PDT. Further research in this field is needed to improve the selectivity and effectiveness of PDT, especially for cancer treatment. The main problem of PS is that approval from medical regulators is always difficult and slow. Nowadays, there are a few approved PSs on the market; some of the most important are summarized in Table 1.

In addition to the aspects discussed in this review, it is essential to consider the photobleaching effect. Photobleaching, the irreversible loss of photosensitizer activity during light exposure, can significantly impact the therapeutic outcome. The phenomenon occurs due to the degradation or damage of the photosensitizer molecule, leading to a decrease in its concentration and consequently limiting the production of ROS crucial for cell destruction. To mitigate the photobleaching effect, various strategies have been explored. These include fractionated light delivery, where the light is administered in multiple sessions with intervals to allow recovery of the photosensitizer. Additionally, optimizing the dosage and formulation of the photosensitizer, as well as employing photostabilizers, have shown promise in reducing photobleaching. Monitoring the kinetics of photobleaching during PDT sessions can also provide valuable insights for treatment optimization. Future studies should focus on developing techniques to mitigate photobleaching and enhance the overall effectiveness of PDT, ensuring optimal clinical outcomes for patients.

In the specific case of skin cancer, the most used for PDT include: ALA and MAL. They are metabolized by cells to produce a photosensitizing agent called protoporphyrin IX (PpIX), which accumulates in cancer cells and is activated by exposure to light. The most relevant trademarks are Photofrin^®^, Levulan^®^, Ameluz^®^, and Metvix^®^; they are described after Table 1 (references are included in Table 1).

- Photofrin^®^ is a mixture of porphyrin-related compounds that is used as a photosensitizing agent in PDT to treat cancer and other medical conditions. It is primarily composed of porfimer sodium, which is activated by red light with a wavelength of 630 nm. When used in PDT, Photofrin^®^ is typically delivered several hours to a few days before the treatment itself. The drug is metabolized in the liver and excreted in the feces and urine. Photofrin^®^ is approved by the U.S. Food and Drug Administration (FDA) for treating certain types of cancer, such as esophageal, lung, bladder, and skin cancer. It is also used to treat Barrett’s esophagus, a pre-cancerous condition of the esophagus. When red light activates, Photofrin^®^ produces ROS that can damage and ultimately kill cancerous and other abnormal cells. The drug is taken up by cancer cells and localizes in the mitochondria and Golgi apparatus. Common side effects of Photofrin^®^ include sensitivity to light, skin reactions, nausea, and vomiting. In rare cases, severe allergic reactions and respiratory distress may occur. Photofrin^®^ is manufactured by Concordia Laboratories Inc. and distributed in the United States by Pinnacle Biologics Inc.
- Ameluz^®^ and Levulan^®^ are both brand names for different formulations of the same active ingredient, 5-aminolevulinic acid (5-ALA). The main difference lies in the vehicle or gel used to deliver the 5-ALA. Ameluz is a gel formulation of 5-ALA approved for the treatment of actinic keratosis, and it is often used with a blue light source for activation. Levulan, on the other hand, is a solution or cream formulation of 5-ALA used for various dermatological conditions, including actinic keratosis and acne. Levulan can be activated with different light sources, such as blue light or intense pulsed light, depending on the indication. Both formulations have been extensively tested and approved for clinical use in several countries, with side effects including skin photosensitivity and discomfort at the treatment site. The clearance and cellular localization of the drugs are similar, with Ameluz having a longer waiting time before treatment and usually being activated by red light, while Levulan has a shorter waiting time and is activated by blue light. Ameluz is manufactured by Biofrontera AG, while Levulan is manufactured by DUSA Pharmaceuticals, Inc.
- Metvix^®^ is a photosensitizing agent used in PDT for the treatment of various types of cancer and pre-cancerous skin lesions. The active substance in Metvix^®^ is MAL, a prodrug converted to PpIX in the presence of light. PpIX then accumulates in the target cells and tissues, leading to their destruction upon exposure to light of a specific wavelength. The key factor contributing to selectivity is the final step in heme synthesis, where ferrochelatase inserts Fe2+ into PPIX. In cancers, this step is often decreased due to reduced ferrochelatase activity and lower iron levels, leading to the observed selectivity. The lambda (nm) of Metvix^®^ is approximately 635 nm, which is in the red light spectrum. The time to PDT after dose delivery is typically around 3 h, although this can vary depending on the specific condition being treated and the individual patient. Metvix^®^ is metabolized and cleared from the body relatively quickly, with most of the drug being eliminated within 24 h. Metvix^®^ has been approved for clinical use in treating AK and superficial BCC. It has also been used off-label for treating other types of cancer, such as lung and bladder cancer. However, more research is needed to fully evaluate its effectiveness in these indications. The most common side effects of Metvix^®^ include skin irritation, redness, swelling at the site of application, and mild to moderate pain or discomfort during and after PDT. More serious side effects, such as skin infection, blistering, and scarring, may occur in rare cases. Metvix^®^ is localized primarily in the target cells and tissues, such as cancerous or pre-cancerous lesions in the skin. Upon exposure to light, the photosensitizing agent produces ROS that induces cell death and tissue destruction in these target areas. The primary mechanism of action of Metvix^®^ is through the production of ROS upon exposure to light, leading to cell death and destruction of the target tissues. The manufacturer of Metvix^®^ is Galderma, a multinational pharmaceutical company specializing in dermatology and skin care products.

These PSs have been used to treat various skin conditions, including actinic keratosis, acne, and some types of skin cancer, among others. Metvix^®^ and Ameluz^®^ are commonly used for the treatment of AK, while Photofrin^®^ and Levulan^®^ have been used for the treatment of BCC and SCC. However, it is important to note that the specific use of each PS may vary depending on the country and the specific medical application.

## 4. Light Sources

The traditional application of PDT has been limited to superficial lesions due to the complexity involved in guiding light into deep target internal organs to activate PSs. One key factor in PDT efficacy is the light source used, which must be suitable for both the target tissue and the PS being employed. In addition to the type of light source, the wavelength of the light and the intensity-modulating pattern of the illuminating beam are also important factors to consider in superficial PDT. The therapeutic window for PDT has typically been defined between 600 and 800 nm for non-superficial treatment [70], while blue light (~400 nm) is used for superficial cutaneous treatment (penetration depth around 1–2 mm), and red light (~650 nm) is used primarily for more profound tissue treatment because it penetrates up to about 1 cm [15]. Wavelengths beyond 800 nm are usually inefficient in promoting an oxygen molecule from the triplet to the singlet state [70] due to insufficient energy (lower than 1.55 eV) of each photon inside the light-radiating beam to excite the PS. The penetration depth of the light is limited by the absorption of light by blood and scattering mechanisms [71,72]. Therefore, the selection of the appropriate wavelength for PDT should be based on the depth of the lesion, the optical properties of the tissue, and the absorption spectrum of the PS. PDT anti-tumor effects can be achieved for various superficial [73] treatment sites using a wide variety of light sources, both coherent and incoherent.

Coherent light sources, particularly lasers, have been widely used in PDT for superficial skin cancers due to their monochromaticity, high power, and efficiency in coupling with optical fibres [70]. The use of lasers in PDT has several advantages over other light sources. One of the main advantages of laser light is its ability to be targeted to specific PSs, making it a suitable option for PDT with a range of PSs. This targeted approach minimizes the damage to healthy tissues and increases the effectiveness of the treatment. Lasers can also deliver high power levels, which allows for a shorter treatment time and greater selectivity in destroying cancerous cells while minimizing damage to surrounding healthy tissue [70,71]. Another advantage of lasers in PDT is their ability to provide uniform irradiance through iris and beam expanders, which is particularly important for achieving a homogenous distribution of light across the treatment area [71]. This uniform distribution of light ensures that all areas of the lesion receive the same amount of light, increasing the effectiveness of the treatment. However, the use of lasers for PDT also has some drawbacks. One of the main drawbacks is that lasers can be challenging to adapt for specific treatment sites and are generally unsuitable for whole-body treatment. This limitation is due to the fact that lasers can only be used on small areas and cannot cover large areas of the body.

Non-coherent light sources, such as lamps and LEDs, serve as cost-effective, versatile alternatives to lasers in superficial photodynamic therapy (PDT) [74]. These broad-spectrum sources can activate a range of photosensitizers (PSs) and offer wide illumination fields for treating larger areas [75]. However, they may provide non-uniform irradiance, leading to uneven treatment [76], and tissue heating, which can cause extended inflammation [75]. LEDs offer several benefits, such as low cost, portability, and specific wavelength emission, making them adaptable for various applications. They can provide high-intensity light for excellent tumor control in superficial lesions. Their long lifetime makes them cost-effective for repeated treatments, and the wavelength of LED light can be adjusted for efficient PS activation while minimizing healthy tissue damage. Incandescent lamps, another non-coherent light source, emit broad-spectrum white light that can be filtered. They are inexpensive and widely available but less efficient at activating PSs than lasers and LEDs, possibly necessitating longer treatment times [77]. Xenon arc lamps also emit broad-spectrum light suitable for treating a range of superficial lesions [70]. They can emit high-intensity light to effectively activate PSs, but they are less portable, more costly, and may need more complex setups.

In summary, choosing a light source for superficial PDT depends on several factors, including the specific treatment site, cost, adaptability, wavelength, and power requirements. Table 2 outlines the fundamental characteristics of each type of source [78]. Coherent light sources, such as lasers, are highly efficient at activating PSs, but they are expensive and less adaptable than non-coherent sources.

Only a few studies have directly compared different light sources, with some reporting similar outcomes [79,80], while others show slightly better results with lasers [81]. Non-coherent sources, such as LEDs and incandescent lamps, are less expensive and more adaptable but have limited light penetration depth. Xenon arc lamps offer a broad spectrum of light and high intensity but are less portable and more expensive. Overall, each light source has its advantages and disadvantages, and the choice of light source should be based on the specific needs of the patient and the nature of the lesion being treated. However, with the impressive advances in laser and LED solid-state technologies, which can be easily modulated in intensity and are equipped with great optical and/or electronic control of fluence rates, both are undoubtedly the preferred technologies for supplying appropriate photon doses for PDT. Technically, laser technology sources offer higher possibilities to implement more accurate, stable, and focalized doses, which are necessary for effective and efficient treatments.

In the realm of skin cancer treatment, different light sources in PDT have shown varying levels of efficacy and suitability. Each light source carries its unique benefits and drawbacks that warrant closer examination, particularly in the context of skin cancer. Lasers, as coherent light sources, have shown promising results in the treatment of BCC, one of the most common types of skin cancer [82]. Their precise targeting capabilities allow for minimal damage to the surrounding healthy tissues while ensuring maximum PS activation within the cancerous cells. On the other hand, non-coherent light sources like LED and lamps have been utilized effectively for the treatment of superficial skin cancers, such as actinic keratosis [83,84,85]. LEDs, with their low cost, specific wavelength emission, and portability, present a viable option for wide-area superficial treatments. Despite their limited penetration depth (in comparison with lasers), multiple LED sources can be strategically placed to achieve satisfactory treatment of deeper lesions (combining different wavelengths). Comparatively, incandescent lamps, while being economical and readily available, might require extended treatment times due to their lower efficiency at PS activation [83]. Similarly, Xenon arc lamps can effectively activate PSs with high-intensity light, but their use may be restricted due to higher cost and less portability [86]. Notably, several studies have highlighted the comparable efficacy of different light sources in treating skin cancers [32,87,88,89,90]. For example, Ref. [32] found that in comparison to other sources, LED treatment outcomes are equal, if not superior, according to some data. However, another study suggested an increased effectiveness of laser-assisted PDT regarding lesion clearance in treating actinic keratosis [90].

In conclusion, the choice of light source in PDT for skin cancer treatment is multifactorial and should be determined based on the specific needs of the patient, the nature of the lesion, and the practical considerations of the treatment setting. Continuous advancements in light technologies, particularly laser and LED, present promising opportunities for enhanced, targeted skin cancer treatment.

## 5. Advanced Techniques and Light Source Technologies

As previously stated, PDT is a highly effective cancer treatment that relies on precise light delivery to a targeted area. The effectiveness of PDT depends on uniform and consistent light delivery to the entire tumor volume without affecting surrounding healthy tissue. The light dose, corresponding to the number of photons the photosensitizer (PS) absorbs per gram of tissue, is influenced by the characteristics of the PS (like its absorption coefficient, quantum yield, and photobleaching rate), the oxygen concentration at the treatment site, and the light applied. The fluence rate and illumination duration are significant as they directly affect the photodynamic dose. The radiant energy fluence rate, which measures the total power incident on an infinitesimally small sphere divided by the sphere’s cross-sectional area, is expressed in W/cm^2^. Moreover, fluence can be defined as the integration of the fluence rate over time, and its units are J/cm^2^. It is crucial to report both the fluence rate and illumination duration, not just the fluence, as the same energy fluence can result in significantly different photodynamic therapy outcomes, depending on the treatment conditions. Light source characteristics, such as coherence, irradiated area, or type of delivery, can influence the dose and efficacy of photodynamic therapy. Recent advancements in PDT technology have overcome traditional light source limitations, such as low irradiance, poor spatial uniformity, and limited penetration depth. These advancements enable optimal therapeutic outcomes and reduce the risk of treatment-related side effects. Novel light source technologies with controlled irradiance, better spatial uniformity, and deeper penetration have been developed to improve the success of PDT. In addition, innovative techniques have been created to enhance light delivery to the targeted area. This section provides a comprehensive overview of the latest developments in PDT technology, including recent advances in light source technologies for skin cancers and innovative techniques for improving light delivery to the targeted area.

### 5.1. Advanced Techniques

Over the years, different techniques have been developed to enhance the efficacy of PDT while minimizing side effects. Four of these techniques are daylight PDT, metronomic PDT, fractioned PDT, and two-photon PDT. This section will explore the principles behind each technique, their advantages and disadvantages, and their potential applications in treating skin cancer.

#### 5.1.1. Daylight PDT

**Figure 2:**
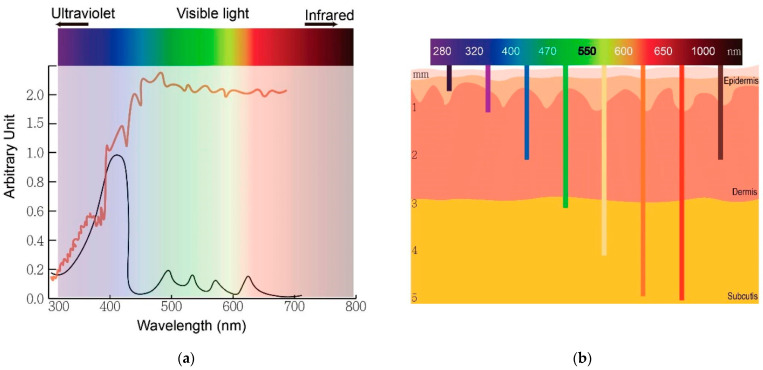
The wavelength of light determines an optimal therapeutic window of PDT. (**a**) The absorption peaks of protoporphyrin IX (black) and sunlight spectrum at sea level (brown). (**b**) The relationship between wavelengths of light and skin penetration depth. Reprinted with permission from Ref. [35]. Copyright year 2020 by the authors. Licensee MDPI, Basel, Switzerland.

Daylight PDT (dPDT) is a relatively new technique for skin cancer treatment that involves the administration of a PS followed by exposure to ambient daylight rather than a specific light source [34,35,36]. Figure 2a shows the broad spectrum of solar radiation and the peak absorption of protoporphyrin IX, whereas Figure 2b shows the relationship between wavelengths of light and skin penetration.

dPDT was first employed clinically in Copenhagen in 2008 for treating skin cancers [35]. The goal of dPDT is to reduce the cost and inconvenience of treatment and increase patient accessibility to PDT. One of the key advantages of dPDT is its simplicity and ease of use. Unlike conventional PDT, which requires specialized equipment and facilities, dPDT can be performed outdoors with access to natural sunlight. This makes it an attractive option for patients living in remote or rural areas with limited access to medical facilities. Additionally, dPDT can be performed relatively quickly, typically requiring only a few hours of sunlight exposure [91]. Daylight PDT has been studied extensively in clinical trials to treat various types of skin cancer, including BCC, SCC, and AK [31,92]. For example, in the latter reference, seventeen publications that explored the application of dPDT were analysed, concluding that the rate of total responses to the treatment of AKs, as reported in randomized trials, varies between 46% and 89.2%. Currently, aminolevulinic-acid-mediated dPDT is utilized for treating multiple or confluent AK skin lesions, using natural daylight as the light source. Specifically, Levulan^®^ Kerastick™, which contains a 20% *w*/*v* ALA solution, is approved by the FDA for managing AK on the face, scalp, and upper extremities, specifically when used alongside a blue light source (400–430 nm) [34]. In the U.K., ALA methyl-ester (MAL) has been approved as Metvix^®^, a 16% *w*/*w* MAL cream utilized for treating thin or non-hyperkeratotic and non-pigmented AK on the face and scalp. Recently, a lecithin-based nanoemulsion with a 10% *w*/*w* ALA concentration was given approval by both the FDA (as BF-200 ALA) and EMA (as Ameluz^®^) for the targeted treatment of mild-to-moderate AK on the face and scalp [93,94]. Contrary to the FDA’s requirement of specifying both a prodrug and a particular light source to be used in combination, the U.K. and Europe allow Metvix and Ameluz to be used with any narrowband or broadband light source [34]. Several randomized trials conducted in Europe and Australia have evaluated the effectiveness and safety of using daylight PDT with MAL to treat AK [95,96,97,98,99]. These trials have included a small exploratory study comparing daylight PDT to conventional PDT. In all trials, participants experienced high total lesion response rates (>70% grade I) with minimal to no pain during daylight exposure. Pain associated with treatment seems to be significantly less than for conventional PDT [100]. Another potential advantage of dPDT is its ability to induce an immune response in addition to the direct cytotoxic effects on cancer cells [101]. The exposure of the skin to natural sunlight during dPDT can stimulate the production of cytokines and other immune modulators, which can help to activate and mobilize the immune system to target and destroy cancer cells [102]. This immune response may also help to reduce the risk of recurrence and improve overall treatment outcomes. However, there are also some challenges associated with dPDT. One major limitation is the variability in sunlight exposure, which can be influenced by various factors, such as cloud cover, latitude, and time of year. For example, when analysing UV exposure during dPDT [91], the maximum average UVE exposure in the UK was found to be 8.2 standard erythemal doses (SED) at Camborne, with a PpIX dose of 23.4 J cm^−2^. Starting treatment earlier in the day leads to a decrease in average UV exposure (Camborne: 5.2 SED, PpIX dose: 18.2 J cm^−2^), while the PpIX dose still reaches the threshold during the winter months (Camborne, November: 0.8 SED, PpIX dose: 7.1 J cm^−2^). In places like Cyprus and Gibraltar, which have high UV exposure during dPDT, the maximum values recorded in June are 14.3 SED and 12.9 SED, with corresponding PpIX doses of 36.1 J cm^−2^ and 35.1 J cm^−2^. UVA exposure data are also included for comparison. This can make it difficult to control and standardize the amount of light exposure received by patients, which may impact treatment efficacy. Additionally, the use of natural sunlight as the light source may increase the risk of photosensitivity and skin damage, which can be significant concerns in patients with fair skin or a history of sunburn. In order to overcome some of these challenges, researchers have explored the use of devices that can standardize and control the amount of light exposure during dPDT (Artificial dPDT).

Artificial dPDT involves the use of light sources that mimic natural daylight, providing a more consistent and reproducible treatment. Several approaches have been developed to obtain uniform light distributions for artificial daylight PDT. One example is the use of artificial white light sources as an alternative to natural daylight for treating AK. In a study conducted by Maire et al. in 2020 [85], a CE-marked device was used to deliver uniform illumination with 2.9 mW·cm^−2^ over a 314 cm^2^ surface. This approach has the potential to improve the efficacy of PDT by providing more consistent and controllable light exposure. In addition to artificial white light sources, other approaches have been developed to obtain uniform light distributions for artificial daylight PDT. Multi-wavelength LED [103] and non-coherent UV-protected greenhouses [104] are just a few examples of these approaches. These technologies offer greater flexibility in terms of light intensity, wavelength, and distribution, which can help to improve the overall effectiveness of PDT. One promising development in the field of artificial daylight PDT is the use of a uniform-illumination light source that can tune the direction of light emission, providing uniformity across large anatomical surfaces, such as the head or leg. This approach, which was implemented by O’Mahoney et al. in 2018 [105], uses LED chips that can independently emit seven distinct wavebands of light. This technology has the potential to improve the efficacy of PDT by providing more precise and consistent light exposure.

Overall, Artificial dPDT is a promising approach for treating skin cancer, offering several potential benefits over conventional PDT. By using natural sunlight as the light source, daylight PDT can reduce the cost and inconvenience of treatment and increase patient accessibility to PDT.

#### 5.1.2. Metronomic PDT

Another approach to improving the effectiveness of PDT is the use of metronomic PDT (mPDT) [106], which induces cancer cell death by intermittent continuous irradiation with a relatively weak power of light for a long duration (days). Metronomic PDT is an established technique that was initially proposed for brain tumors, where maintaining a balance between tumor cell destruction and the preservation of healthy brain tissue is essential [107]. Although it has primarily been investigated for brain tumors, it has also been explored in recent years as an alternative to conventional PDT for the treatment of skin cancer [101,108,109]. This technique involves the repeated administration of low doses of the PS, often daily or weekly, rather than a single high dose. One advantage of metronomic PDT is that it can improve treatment outcomes and reduce side effects compared to conventional PDT [110]. The low doses of PS used in metronomic PDT may reduce the risk of photosensitivity and skin damage, which can be significant concerns in conventional PDT. Additionally, prolonged exposure to ROS may increase the likelihood of inducing cell death in tumor cells and decrease the chance of developing resistance to treatment [111]. Metronomic PDT has been studied extensively in preclinical models, with promising results. In one study, mice bearing melanoma tumors were treated with either conventional or metronomic PDT. The metronomic PDT group showed a significant reduction in tumor growth and increased survival compared to the conventional PDT group [109]. Another study found that metronomic PDT induced immune activation and subsequent regression of tumor lesions in murine models of skin cancer and pre-cancer [108]. However, there are also some challenges associated with metronomic PDT. One major limitation is the need for repeated treatments over a prolonged period of time, which can be inconvenient and increase the overall cost of treatment. Additionally, the optimal dosing schedule and duration of treatment have not yet been established, and may vary depending on the type and stage of cancer being treated.

Metronomic PDT is a promising approach for treating skin cancer, offering several potential benefits over conventional PDT. By maintaining a constant level of the PS in tumor tissue, metronomic PDT may lead to improved treatment outcomes and reduced side effects. While some challenges are still associated with this technique, ongoing research is focused on advanced light delivery sources that allow the dose optimization to maximize its effectiveness.

#### 5.1.3. Fractioned PDT

Fractioned PDT is also an old technique that involves the administration of the photosensitizing drug in multiple small doses over some time, rather than a single high dose (the discrete version of metronomic PDT) [112,113]. This approach has been shown to reduce the risk of skin photosensitivity. It may be more effective against larger tumors, making it a promising technique for treating skin cancer [114]. In traditional PDT, a single high dose of the photosensitizing drug is administered, followed by exposure to a light source. While effective, this approach can be associated with significant side effects, including pain, swelling, and skin photosensitivity. Fractioned PDT, on the other hand, involves the administration of multiple small doses of the photosensitizing drug over some time, reducing the risk of side effects. Several studies have demonstrated that fractionated ALA-PDT is superior to conventional illumination for the treatment of AK (94% vs. 85% at 1 year) and superficial BCC (sBCC) (88% vs. 75% at 5 years), but not for SCC in situ (88% vs. 80% at 1 year) [115,116,117]. A large series of 552 lesions (including AK, SCC in situ, sBCC, and nodular BCC) treated with ALA-PDT using two light fractions of 20 and 80 J/cm^2^ at 4 and 6 h, separated by a 2 h dark interval, reported an overall clearance rate of 95% after a 2-year follow-up [118]. An alternative fractionation protocol using two doses of 75 J/cm^2^ at 4 and 5 h was initially associated with a 94% clearance rate for nodular BCC, but a cumulative failure rate of 30% was observed after 3 years [119]. A study of 162 patients with sBCC found no significant difference in efficacy between standard red light MAL-PDT and fractionated ALA-PDT, which was attributed to differences in agent localization [120]. Fractioned PDT also showed a favorable safety profile, with no serious adverse events reported. The most common side effects were erythema (redness of the skin), edema (swelling), and hyperpigmentation (darkening of the skin), which were mild to moderate in severity and resolved within a few weeks after the treatment [121]. Another advantage of fractioned PDT is its potential to improve cosmetic outcomes compared to traditional PDT. Because the photosensitizing drug is administered in multiple small doses, this reduces the risk of scarring or other cosmetic complications. While promising, fractioned PDT is not without its limitations. The treatment requires multiple sessions over several weeks, which can be inconvenient for some patients. Additionally, the cost of fractioned PDT may be higher than traditional PDT due to the need for multiple treatments, and, in some cases, it has allowed for tumor resistance and clinical relapse [106]. However, the potential benefits of improved efficacy, reduced side effects, and improved cosmetic outcomes may make fractioned PDT a worthwhile option for patients with skin cancer.

Fractioned PDT is a promising technique for treating skin cancer, potentially improving efficacy and reducing the risk of side effects compared to traditional PDT. While further research is needed to fully evaluate the long-term efficacy and safety of fractioned PDT, the results of recent studies suggest that it may be a viable treatment option for patients with SCC or BCC.

#### 5.1.4. Two-Photon PDT

**Figure 3:**
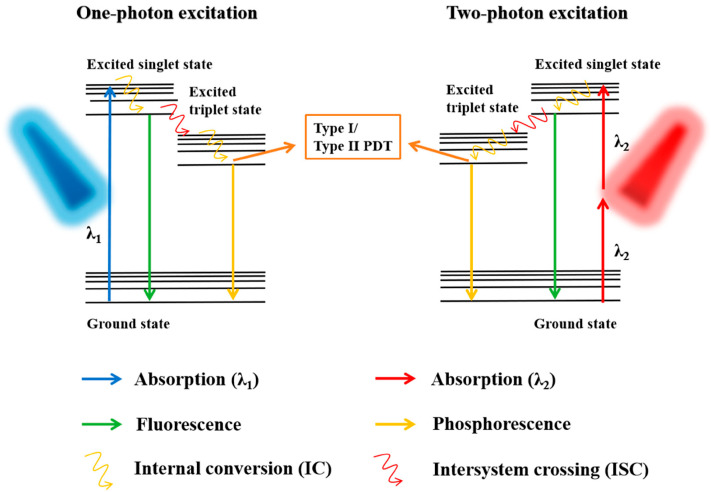
Photosensitizer activation through one-photon excitation and two-photon excitation (TPE). The TPE requires two photons of approximately half the energy compared to the energy needed in one-photon excitation. Reprinted with permission from Ref. [44]. Copyright year 2021 by the authors. Licensee MDPI, Basel, Switzerland.

Two-photon PDT (TP-PDT) is also an old technique [122] that has been developed over the last years thanks to new laser sources and PS. This technique is based on the use of two photons of near-infrared (NIR) light that are absorbed simultaneously by a PS molecule, resulting in the generation of singlet oxygen and other ROS that can kill cancer cells (see Figure 3) [44].

One of the main advantages of TP-PDT is its ability to penetrate deeper into the skin than traditional PDT techniques. This is because the two-photon absorption process is highly localized, which allows for precise targeting of cancer cells without damaging surrounding healthy tissue [123]. In addition, the use of NIR light reduces the risk of phototoxicity and other adverse side effects associated with traditional PDT, as it has lower energy and longer wavelengths that can penetrate deeper into the skin. TP-PDT has been shown to be effective in the treatment of a variety of skin cancers, including BCC, SCC, and melanoma [124,125]. One of the challenges of TP-PDT is the need for specialized equipment, including a femtosecond laser, which can make the technique more expensive and less accessible than traditional PDT. However, technological advances have made these systems more widely available, and the potential benefits of TP-PDT in the treatment of skin cancer make it a promising area of research. Another advantage of TP-PDT is its ability to be combined with other treatment modalities, such as immunotherapy and chemotherapy. This combination therapy approach has been shown to enhance the therapeutic efficacy of TP-PDT and improve patient outcomes. Despite its potential advantages, there are also some limitations to TP-PDT. One of these limitations is the need for a PS molecule that can absorb two photons of NIR light, which limits the range of PSs that can be used with this technique [126]. Another challenge of TP-PDT is the lack of standardized protocols and dosimetry, making it difficult to compare results across studies and optimize treatment parameters [127]. However, ongoing research is focused on addressing these issues and improving the effectiveness and accessibility of TP-PDT for treating skin cancer and providing new PS [128].

In conclusion, TP-PDT is a promising technique for treating skin cancer with potential advantages over traditional PDT techniques. Its ability to penetrate deeper into the skin, its higher capacity for spatial focalization, and its compatibility with other treatment modalities make it an attractive option for patients with skin cancer. However, further research is needed to optimize treatment protocols, improve accessibility, and provide new PS for this technique.

### 5.2. Advanced Light Delivery Devices

Advanced light delivery devices have revolutionized the field of medicine, particularly in the realm of PDT. In this specific case, the efficacy largely depends on the ability to administer the therapeutic light specifically to the entire area of the target tissue. For PDT to be most effective and to minimize harm to surrounding tissues, the treatment target should be evenly illuminated by a well-conforming light source. This uniformity can be achieved by attaching a diffusing cover to the end of the optical fibers. This technique is highly suitable for treating easily accessible flat surfaces and has been widely used in PDT for skin lesions [70]. Optical fibers are essential components of most flexible light delivery devices used in skin cancer treatments. Diffusing tips, produced by companies like SCHOTT, Medlight, Pinnacle Biologics, and Biolitec, facilitate an even distribution of light along the diffuser’s length. This is achieved by introducing surface roughness or scattering particles at the border between the core and cladding of the fibers. While standard diffusers generate uniform illumination throughout their length, custom diffusers can be tailored to produce a light profile that aligns with particular treatment plans [129]. In this regard, optical fibers with diffusers are usually employed in advanced light delivery devices as light-emitting fabrics. Plastics and adhesives are also some of the latest innovations in this domain. These materials offer unique advantages over traditional light delivery methods by providing more flexibility, comfort, and better patient outcomes. In this section, we will only be focused on and delve deeper into the potential benefits of light-emitting fabrics, plastics, and adhesives for PDT, and explore how they are being used to improve treatment outcomes for skin cancer.

#### 5.2.1. Light-Emitting Fabrics

Light-emitting fabrics [39] are promising technologies for delivering light-based therapies, such as PDT. Textile light diffusers are a type of fabric that can diffuse light in a controlled manner. These fabrics are made of various materials, such as nylon or polyester, that can be treated with a special coating to make them light-diffusing. These diffusers can be made into various shapes and sizes and placed directly on the skin or inserted into the affected area. They can also be customized to match the shape of the affected area, ensuring that the light is delivered precisely where it is needed. Typically, the light source is generated using optical fibres that have been manipulated to disperse light through their sides instead of the end-face (typical macrobending). Macrobending of plastic optical fibre (POF) can be induced by the specific architecture of textile structures, resulting in side-emission without the need for any post-treatment. The bending angle influences the degree of light decay along the fibre, and the calculations for this phenomenon are elaborated upon by Endruweit et al. [130]. The latter study uses the Monte Carlo ray-tracing method to examine how light behaves in optical fibres, particularly those with a single bend. It shows that a smaller critical angle for total internal reflection and a smaller light beam opening angle increase light guidance efficiency. Uniform distribution of light across the fibre face yields higher transmission, which decreases with bending angles. Major light emission zones that emerge with increased bending angles cause noticeable transmission losses. However, such losses drop exponentially with an increased ratio of bending radius to fibre radius. Macroscale bending in composite components has a minimal impact on light transmission, but mesoscale bends can cause significant losses. The study also found that total transmission losses across multiple fibre bends differed from previous estimates, as passing through a bend could change the distribution of light within the fibre. Fabrics with a lower degree of crimp were found to be most suitable for optical fibre integration due to reduced bending losses in light intensity. Embroidery or weaving techniques can be utilized to create the macrobending necessary for this effect.

**Figure 4:**
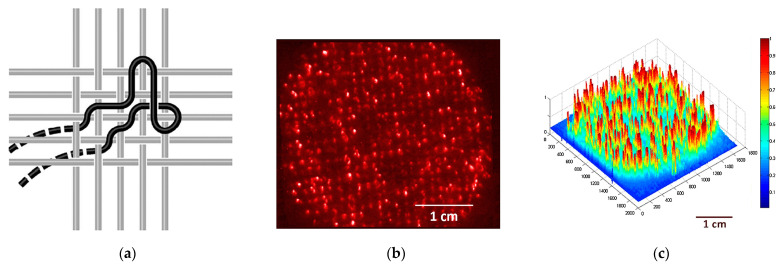
(**a**) Schematics of an embroidery-based light-emitting fabric, (**b**) embroidery-based light-emitting fabric developed by Selm et al. [131], and (**c**) light distribution of this embroidery-based light-emitting fabric. Uneven light distribution is observed. Reprinted with permission from Ref. [37]. Copyright © 2016 Elsevier Ltd.

In the first case, the embroidery process enables the creation of various patterns on POF, including spots, lines, curves, and zigzags, on various substrate materials (see Figure 4a). The use of embroidery to create these patterns on POF has several advantages. Firstly, it allows for precise control over the placement and shape of the pattern, which is important for ensuring that the light is delivered to the targeted cells effectively. Secondly, embroidery is a relatively inexpensive and straightforward process, so it can be easily scaled up for large-scale production. Selm et al. evaluated this technique by developing a prototype consisting of a dense woven substrate with embroidered POF fixed using conventional yarn (Figure 4) [131]. The woven substrate was made of 100% polyester multifilament, weighing 50 g/m^2^ and 29 threads cm^−1^ in warp and weft. This approach, in combination with an aluminium backing and embroidered textile, provides adequate irradiation power for low-fluence-rate (3.6 mW cm^−2^) PDT treatment over a large area of several square centimeters (11 cm^2^).

On the other hand, weaving can be used to address the issue of reduced side-emitted radiation intensity along the fibre and to enhance the uniformity of light emission. Weaving is a well-known textile production technique that has been used for centuries to produce fabrics of various types. It is a process that involves interlacing two different sets of yarns or threads at a perpendicular angle to produce a cloth or fabric (Figure 4a). In the case of woven-based light-emitting fabrics, this technique is used to weave POF into a flexible fabric, which can emit light from its sides, providing the highest fluence rate and the best homogeneity of light delivery (see Figure 4b,c). One of the advantages of woven-based light-emitting fabric is its flexibility, which allows it to conform to the contours of the treatment area. This means that it can be used to treat various body parts, including curved and irregularly shaped ones. This flexibility is crucial in delivering light-based therapies, such as PDT, where the light needs to be delivered precisely to the affected area. Additionally, woven-based light-emitting fabric is highly durable, making it suitable for use in medical settings where equipment must be able to withstand frequent use. Another advantage of woven-based light-emitting fabric is its ability to enhance the uniformity of light emission. In traditional light-emitting fabrics, the intensity of light emitted along the fibre decreases as it moves away from the light source. However, the weaving process enhances the uniformity of light emission with woven-based light-emitting fabric. The fibre is bent at various angles, which causes light emitted from the sides, resulting in a more uniform distribution of light. This uniformity of light emission is essential for delivering effective light-based therapies, such as PDT. The weaving process used in woven-based light-emitting fabric is also highly customizable. Various weaving patterns can be used to produce fabrics of different shapes, sizes, and densities, which can be tailored to meet the specific requirements of a particular application.

**Figure 5:**
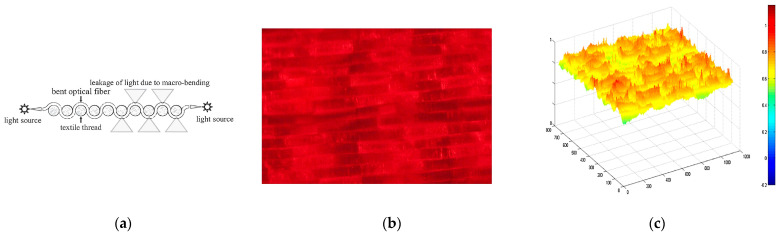
(**a**) Schematics of a woven-based light-emitting fabric, (**b**) woven-based light-emitting fabric, and (**c**) light distribution of woven-based light-emitting fabric. Homogeneous light distribution is observed. Reprinted with permission from Ref. [38]. Copyright © 2014 Elsevier B.V.

Another device that has gained attention is the textile light diffuser developed by Cochrane et al. in 2013 (Figure 5) [132]. The device is based on commercial polymer optical fibres that are arranged in a weave pattern, and it is designed to produce a large diffuser with a useful width of 20 cm. The light source used in this device is a 5 W laser diode that generates a quasi-homogeneous intensity of 18.2 mW·cm^−2^. When considering the use of PDT in direct contact with skin, it is important to be mindful of potential temperature elevations that could cause damage. Infrared measurements taken on the surface of the device indicate that even after 10 min of continuous utilization with a 5 W laser light source, the temperature elevation on the surface is only 0.6 °C. Another advantage of this device is its ability to tune the light source’s wavelength using the same diffuser. This feature allows for flexibility in the wavelength of light used for PDT and can be particularly useful for treating different types of medical conditions.

**Figure 6:**
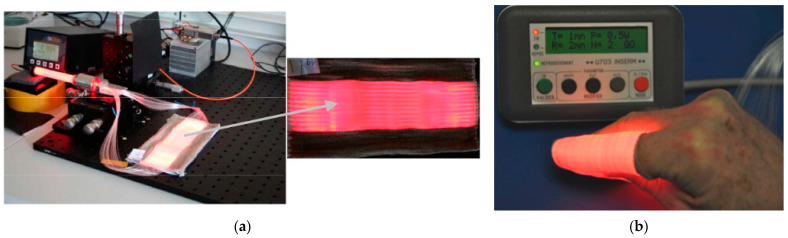
Textile light diffuser (**a**) connected to diode laser light source and (**b**) applied on a finger curved surface. Reprinted with permission from Ref. [132]. Copyright © 2012 Elsevier B.V.

Similarly, the flexibility of the light diffuser used in PDT should be considered to ensure that the light dose received by the curved skin surface is homogeneous. Ideally, a light diffuser that can adapt to the body’s curves should be used. In the wrap direction, the device can be rolled without causing any damage because no POFs are bent. The bend radius is incredibly small, ranging from 1 to 2 mm. The bend radius can be estimated to be between 4 and 5 mm in the weft direction. Figure 6b demonstrates that the device can be placed around a finger without damage, thanks to its flexibility and adaptability. This approach eliminates the need for any pre- or post-treatment of optical fibres, as the weaving parameters alone are sufficient for light diffusion. This significantly impacts the cost of the product, as it utilizes low-cost POF compared to other technologies [132].

Overall, embroidery and woven-based light-emitting fabrics are promising technologies for delivering light-based therapies, such as PDT. In the first case, embroidery allows for precise control over the placement and shape of the pattern. In addition, it is a relatively inexpensive and straightforward process, so it can be easily scaled up for large-scale production. On the other hand, woven-based light-emitting fabrics involve weaving POF into a flexible fabric, which emits light from its sides, providing a high fluence rate and homogenous light delivery. These devices can also be tuned to different wavelengths of light, making them flexible and useful for treating different medical conditions. Finally, using embroidery and woven-based light-emitting fabrics in PDT eliminates the need for pre- or post-treatment of optical fibres, reducing the cost of the product. As these technologies continue to develop, they may become an increasingly important tool in the field of cancer and other disease treatments.

Although light-emitting fabrics are a promising technology, they are still emerging. For this reason, the number of clinical trials using light-emitting fabrics is limited. In [133] (NCT03076892), the authors explored the use of a flexible light-emitting fabrics method (FLEXI-PDT) in treating actinic keratosis, with a specific protocol called Phosistos-PDT (P-PDT) comparing its efficacy to conventional PDT (C-PDT) with a LED panel (Aktilite^®^). The experimental setup included three flexible light-emitting fabrics, each measuring 21.5 × 5 cm and covering a combined illumination area of 322.5 cm^2^. This setup was sequentially exposed to 635 nm laser light at a fluence rate of 12.3 mW cm^−2^ for 1 min, alternating between 1 min of light and 2 min of darkness. The total irradiation time spanned 2.5 h, delivering an overall light dosage of 37 J/cm^2^. The clinical trial consisted of 46 patients treated with P-PDT on one area (n = 285 AK) and with C-PDT on the contralateral area (n = 285 AK). Consequently, [83] compared the FLEXI-PDT to a C-PDT with the same lamp. During the clinical trials (NCT03076918), 156 actinic keratosis cases (42.3% grade I and 56.4% grade II) were treated using the FLEXI-PDT, while 154 cases (42.2% grade I; 56.5% grade II; 1.3% grade III) were subjected to the conventional PDT treatment. A follow-up after three months showed a total response rate of 59.1% for C-PDT and a slightly higher rate of 66.0% for FLEXI-PDT. The six months post-treatment evaluation report response rates increased to 76.8% for C-PDT and 84.0% for FLEXI-PDT, representing a 1.3-fold increase compared to the three-month mark [39]. Both methods induced local side effects, such as erythema and edema, yet they did not necessitate any special care in the context of local dermatological photodynamic therapy. Importantly, patients reported significantly less pain during the FLEXI-PDT treatment. In summary, the FLEXI-PDT is not inferior in terms of efficacy and is superior in terms of tolerability to the conventional protocol for treating AKs of the forehead and scalp. In another clinical evaluation (NCT03076892) the authors used an improved version of the previous device, incorporating the light-emitting fabrics inside an ergonomic helmet [83]. The clinical protocol was similar to the previous one, but with reduced irradiance and light dose. The redesigned device was more ergonomic and compact. It was classified as an exempt risk group device. The results showed that the new PDT technique, called P-PDT, was noninferior to the C-PDT in terms of complete lesion response rate at 3 and 6 months. Specifically, at the 3-month follow-up, for P-PDT, the complete lesion response rate was 79.3%, while for C-PDT, it was 80.7%. Similarly, at the 6-month follow-up, P-PDT achieved a complete lesion response rate of 94.9%, compared to 94.2% for C-PDT. P-PDT resulted in significantly less pain compared to C-PDT during both the first and second treatment sessions. Adverse effects were reported by almost all patients, but the incidence was lower with P-PDT. Overall, P-PDT showed promising results with reduced pain and comparable efficacy to C-PDT in treating actinic keratosis. Finally, another clinical trial that is expected to conclude in August 2024 (NCT03713203) has focused on the rare skin disorder Extramammary Paget disease of the vulva (EMPV), primarily affecting postmenopausal Caucasian females. Surgical excision is the standard treatment, but it often has positive margins and fails to control the disease. Photodynamic therapy using Metvixia has shown promise but is painful. The authors propose a new device based on light-emitting fabrics called PAGETEX [134]. This device was specifically designed to accommodate different body shapes and ensure uniform light distribution in the vaginal area, beneath the labial region, and on the perianal region with safety. The study aims to investigate the efficacy and safety of the PAGETEX medical device, a painless PDT device, in treating vulvar Paget disease. The study is a nonrandomized, single-centre trial with 24 patients. The treatment involves two PDT sessions using PAGETEX at 15-day intervals. Metvixia is applied for 30 min, followed by 2 h and 30 min of red light illumination. The primary endpoint is the disease control rate at 3 months, while secondary endpoints include disease control rate at 6 months, patient quality of life, pain levels during PDT sessions, presence of protoporphyrin IX in Paget cells, and patient satisfaction.

#### 5.2.2. Plastics and Adhesives

Plastics and adhesives have emerged as promising materials for advanced light delivery devices in PDT. These innovative technologies offer a range of benefits over traditional light delivery methods, such as improved flexibility, enhanced patient comfort, and better precision in targeting tumor sites. This section will examine the use of plastics and adhesives in PDT, explore their advantages and limitations, and discuss the latest developments in this rapidly evolving field. From implantable devices to topical applications, plastics and adhesives are transforming how PDT is delivered and providing new hope for patients with various medical conditions.

**Figure 7:**
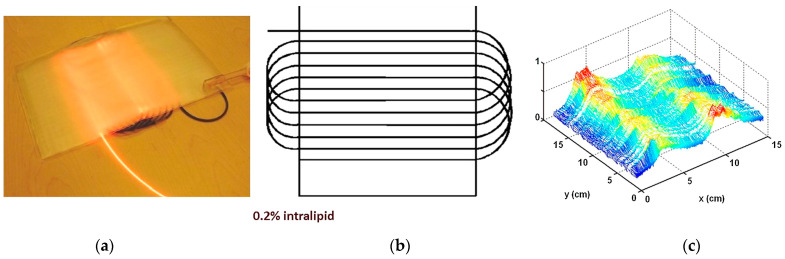
(**a**) Picture of a blanket prototype, (**b**) fibre pattern of light blanket design, and (**c**) light distribution of a blanket prototype (0.2% intralipid; fibre 400 μm; length: 10 m). Uneven light distribution is observed. Reprinted with permission from Ref. [38]. Copyright © 2014 Elsevier B.V.

For example, in [135,136], a light blanket integrating side-glowing optical fibres is demonstrated (Figure 7).

This technique integrates the fibre into a flexible, blanket-like structure that can be draped over the treatment area. The fibres emit light from their sides, illuminating the entire treatment area. Specifically, in this case, the light source is composed of a series of parallel cylindrical diffusing fibres that offer more uniform light delivery and reduced operating time in the operating room. To test its effectiveness, a preliminary experiment was conducted using a 9 cm × 9 cm light blanket consisting of 8–9 cm cylindrical diffusing fibres placed in parallel finger-like pockets. The blanket was filled with a 0.2% intralipid scattering medium to improve the evenness of light distribution. In subsequent work, this parameter was improved by using an optical adaptor, which will be able to match the numerical aperture of the laser source to the numerical aperture of the blanket fibre, thus transmitting a higher percentage of light [137].

**Figure 8:**
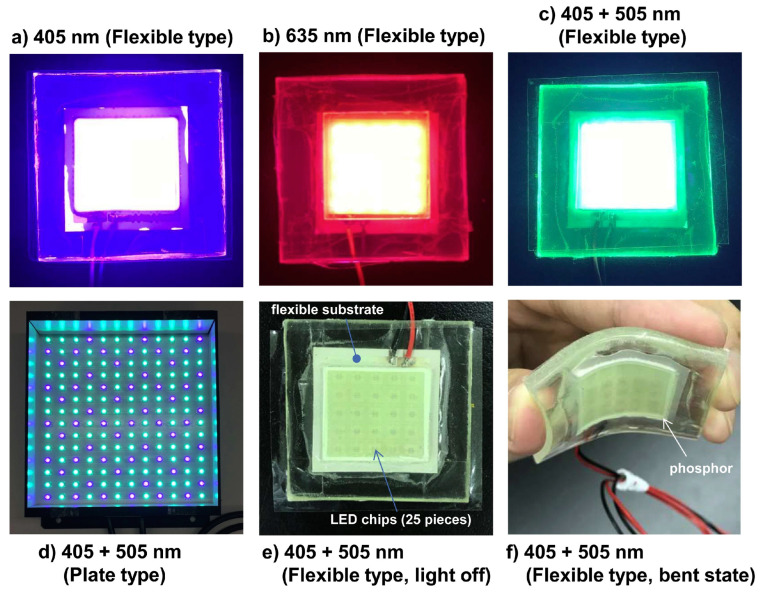
Irradiation devices. (**a**) 405 nm (flexible type), (**b**) 635 nm (flexible type), (**c**) 405 + 505 nm (flexible type), (**d**) 405 + 505 nm (plate type), (**e**) 405 + 505 nm (flexible type, light off), and (**f**) 405 + 505 nm (flexible type, bent state). Reprinted with permission from Ref. [138]. © 2019 Japanese Society for Investigative Dermatology. Published by Elsevier B.V.

On the other hand, flexible materials, such as plastics, can be bent or shaped to match the contours of the affected area. They can also be customized to emit light of a specific wavelength, ensuring that the photosensitizing agent is activated in a targeted manner. They can also be combined with other medical devices, such as endoscopes, to deliver light to areas that are difficult to reach. Masuda et al. manufactured a flexible LED unit in 2019 (Figure 8) [138], which was designed for the multi-wavelength excitation of 5-ALA. This device has the potential to enhance the therapeutic effects of PDT by achieving more uniform irradiation of even areas. With the ability to excite multiple wavelengths, the flexible LED unit can improve the specificity and selectivity of the PS and minimize the damage to normal tissue. This study also investigated the synergistic effects of dual-wavelength irradiation for PDT.

They found that combining 405 nm and 505 nm light produced the greatest synergistic effects in HaCaT cells due to high ROS production. Moreover, the combination of 405 nm and 505 nm light was also the most effective in vivo, as tested on melanoma cells in mice. To achieve the same effect as 405 nm alone, long wavelength light was added to reduce the amount of the shorter wavelength. The level of total ROS at 405 nm + 505 nm was also significantly higher than that of the control and comparable with 405 nm alone. These findings suggest that the combination of 405 nm and 505 nm light produced the greatest synergistic effects in HaCaT cells due to high ROS production. The study also addressed the issue of pain, which is a side effect of PDT. Various methods, such as cooling, topical anesthetics, and lower irradiance, have been used in clinical trials to reduce pain. Using a flexible-type light source unit may help reduce pain, as treatment at home and at a hospital is possible. Irradiation duration may also be increased because treatment can be conducted while performing other activities, and thus low irradiance becomes possible, which might reduce pain.

In 2019, Yamagishi et al. reported a tissue-adhesive optoelectronic device that can deliver PDT wirelessly and continuously to tumors for at least 10 days [139]. The device uses bioadhesive and stretchable PDA-PDMS nanosheets to fix it to internal tissues, allowing continuous delivery of light to target tumors, with negligible risk of thermal tissue damage. The light power used was 1000 times less intense than that of conventional PDT (<100 µW·cm^−2^), ensuring the safety of the device. The researchers overcame the penetration depth limitation of the light by encapsulating the wireless optoelectronics with the PDA-PDMS nanosheets and demonstrated the superiority of green light over red light in treating tumors. The PDA-PDMS nanosheets were fabricated through a gravure-printing method and a PVA sacrificial layer technique and showed significant tissue adhesiveness, making them suitable for stable long-term implantation of small wireless devices in the wet conditions inside the body. The implantable and wirelessly powered mPDT system using tissue-adhesive optoelectronics can be used as an alternative local treatment for tumors in fragile or delicate organs where surgical operation or radiation therapy risks damaging the surrounding healthy tissues, nerves, and blood vessels. The researchers anticipate that the system will offer a new path to treating undetectable microtumors or deeply located lesions where light does not reach by combining the device with the immunostimulating effect of PDT. Future work should focus on developing a light source that can provide broad photoirradiation and flexibly fit the shape of target organs to treat diffusely and widely distributed lesions. An organic electroluminescence device is a promising candidate for this application.

In this regard, other approaches have proposed using deformable organic light-emitting diodes, such as Quantum-LED (QLED) and Organic-LED (OLED). QLEDs have the ability to adjust emission wavelengths and exhibit flexibility, which may facilitate the development of wearable, targeted photomedicine that enhances the absorption of various medical photosensitizers [140,141]. Even though QLEDs have been known to utilize higher power per voltage compared to OLEDs, in the case of PDT applications, experiments involving QLEDs have been conducted at intensities of 5 mW/cm^2^ or lower, which is the same as that used for OLEDs, due to concerns regarding operational reliability [142]. In [143], the authors showed the potential of administering a single dosage of ALA-PDT at a low-fluence rate (3 mW/cm^2^) over an extended treatment duration (3.7 h) using OLED, without requiring the anaesthesia of the animals. The outcome of animals treated with PDT, when monitored for both pre- and post-light tumor volume, is encouraging, as it leads to a greater survival time compared to that of the control group. In [144], an open pilot study of ambulatory PDT using a wearable low-irradiance OLED light source in the treatment of nonmelanoma skin cancer is presented, demonstrating the potential use of this technology over an area of 2 cm in diameter. The study recruited 12 patients with Bowen’s disease or superficial BCC and administered two treatments of red light following the application of ALA. At the 12-month follow-up, seven of the patients remained clear. Pain during and after treatment was scored as <2 using the numerical rating scale. The study suggests that OLED-PDT is less painful and more convenient than conventional PDT, with potential for “home PDT”, but further validation is needed in larger studies.

**Figure 9:**
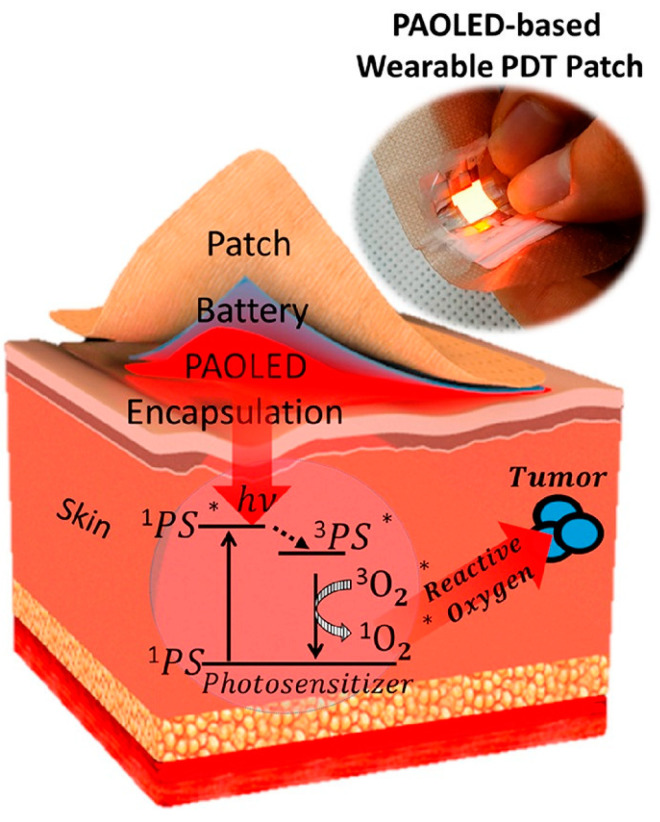
Schematic illustration of PDT treatment principle and a photo of the PAOLED-based wearable PDT patch. * means excited estate. Reprinted with permission from Ref. [142]. Copyright © 2020 American Chemical Society.

An advanced device can be found in [142], in which a parallel-stacked flexible OLED (PAOLED) for wearable PDT is presented (Figure 9).

The article discusses the development of PAOLED for wearable biomedical and display applications. The PAOLED achieved high-output characteristics of over 100 mW/cm^2^ at a low voltage of 8 V or less, with the ability to drive N OLEDs in parallel. The PAOLED was optimized for a specific purpose by adjusting the ITO OLED’s and microcavity OLED’s thickness and current dispersion. It demonstrated an operating reliability of over 100 h even at a high power of 35 mW/cm^2^. For the wearable PDT application, the PAOLED was controlled to match the 660 nm peak wavelength of the photosensitizer and showed the ability to generate about 380% more singlet oxygen than the reference after only 35 min of irradiation under high-power conditions of 35 mW/cm^2^. It was applied in vitro to treat melanoma skin cancer under the same conditions, reducing cell viability by about 24%.

**Figure 10:**
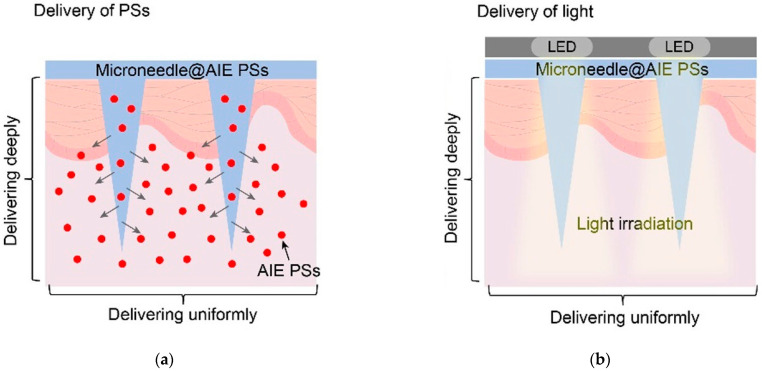
(**a**) Uniform and deep intratumoral delivery of the photosensitizer achieved through microneedles. (**b**) Microneedles and LED arrays used for uniform, deep intratumoral light delivery. Reprinted with permission from [109]. Copyright © 2023 American Chemical Society.

Another interesting recent approach is using a flexible microneedle-based delivery system (Figure 10) [109]. The study used this system to deliver an aggregation-induced emission PS with strong photobleaching ability deep into tumors. The system, called Microneedles@AIE PSs, provided uniform delivery of the PSs, and LED microarray delivered light deep into the tumor through the channels formed by microneedle punctures. Wireless power transmission was used to provide stable power for the light source, allowing treated mice to maintain a good range of motion during the microneedle-based mPDT (M-mPDT) treatment. The mPDT treatment significantly inhibited tumor growth, and it can be used in combination with surgical treatment or immunotherapy to enhance its efficacy. The microneedle-based delivery system has the potential for clinical translation and can improve the antitumor efficacy of mPDT [109].

Overall, plastics and adhesives have become promising materials for advanced light delivery devices in PDT. The use of plastics and adhesives in PDT offers several advantages over traditional light delivery methods, such as improved flexibility, enhanced patient comfort, and better precision in targeting tumor sites. Plastics can be bent or shaped to match the contours of the affected area and can also be customized to emit light of a specific wavelength, ensuring that the photosensitizing agent is activated in a targeted manner. Furthermore, the utilization of deformable organic light-emitting diodes provides the capability to tune emission wavelengths and displays superior flexibility, thereby potentially facilitating the creation of personalized, wearable photomedicine. By utilizing adhesives, treatment can be administered with increased comfort for the patient, also allowing for advanced techniques like metronomic PDT. The use of plastics and adhesives in PDT is transforming the way PDT is delivered and providing new hope for patients with a variety of medical conditions.

## 6. Discussion

Photodynamic therapy is a promising treatment option for various types of skin cancer, which works by selectively destroying cancer cells using photosensitizing agents and light. The mechanism of action of PDT is complex. It involves a series of chemical reactions that result in the generation of ROS when the PSs are activated by light of a specific wavelength. While there are many potential PSs for PDT, only a few have been approved for use in treating skin cancer or other conditions. These PSs have been used to treat various skin conditions, including AK, BCC, and SCC, among others. For example, Metvix^®^ and Ameluz^®^ are commonly used for the treatment of AK, while Photofrin^®^ and Levulan^®^ have been used for the treatment of BCC and SCC. The rigorous testing and regulatory approval process that new drugs and therapies must undergo before they can be marketed to the public partly explain why only a few have been approved.

The choice of light source for PDT depends on several factors, including the specific treatment site, cost, adaptability, wavelength, and power requirements. Coherent light sources, such as lasers, are highly efficient at activating PSs, but they are expensive and less adaptable than non-coherent sources. Non-coherent sources, such as LEDs and incandescent lamps, are less expensive and more adaptable, but they have limited light penetration depth in comparison with lasers. Xenon arc lamps offer a broad spectrum of light and high- intensity, but they are less portable and more expensive. Each light source has its advantages and disadvantages, and the choice of light source should be based on the specific needs of the patient and the nature of the lesion being treated.

Advanced techniques in PDT, such as artificial dPDT and mPDT, offer several potential benefits over conventional PDT. By using natural sunlight as the light source, daylight PDT can reduce the cost and inconvenience of treatment and increase patient accessibility to PDT. In addition, mPDT, which maintains a constant level of the PS in tumor tissue, may lead to improved treatment outcomes and reduced side effects. The discrete version of this technique, fractioned PDT, also improves efficacy and reduces the risk of side effects compared to traditional PDT. While further research is needed to fully evaluate the long-term efficacy and safety of fractioned PDT, the results of recent studies suggest that it may be a viable treatment option for patients with SCC or BCC. Ultimately, TP-PDT shows promise as a technique for treating skin cancer, potentially offering benefits over conventional PDT methods. Its ability to penetrate deeper into the skin and be used in conjunction with other treatments makes it an appealing choice for those with skin cancer. However, additional research is necessary to refine treatment protocols, increase accessibility, and develop new photosensitizers for this approach.

Moreover, advanced light delivery devices in PDT, such as textile light diffusers, woven-based light-emitting fabrics, and plastics and adhesives, have shown great promise. These technologies offer several advantages over traditional light delivery methods, such as improved flexibility, enhanced patient comfort, and better precision in targeting tumor sites. On the one hand, embroidery and woven-based light-emitting fabrics are promising technologies for delivering light-based therapies, such as PDT. Embroidery allows for precise control over the placement and shape of the pattern, while woven-based light-emitting fabrics involve weaving POF into a flexible fabric, which emits light from its sides. These devices can be tuned to different wavelengths of light, making them flexible and useful for treating different medical conditions. The use of these technologies in PDT eliminates the need for pre- or post-treatment of optical fibres, reducing the cost of the product. On the other hand, plastics and adhesives have become promising materials for advanced light delivery devices in PDT. They offer several advantages over traditional light delivery methods, such as improved flexibility, enhanced patient comfort, and better precision in targeting tumor sites. The utilization of deformable organic light-emitting diodes provides the capability to tune emission wavelengths and displays superior flexibility, potentially facilitating the creation of personalized, wearable photomedicine. By utilizing adhesives, treatment can be administered with increased comfort for the patient, also allowing for advanced techniques like metronomic PDT. This approach is transforming the way PDT is delivered and providing new hope for patients with a variety of medical conditions. Even though advanced light delivery mechanisms in PDT have shown promising results, more time and evidence are needed to comprehensively evaluate their advantages and disadvantages.

On the other hand, the standardization of light source equipment in clinical PDT for skin cancer is a challenging yet critical endeavor. This would significantly contribute to ensuring consistent and optimal therapeutic outcomes across different healthcare facilities. Key factors to consider include the wavelength, power, beam profile, irradiance uniformity, and penetration depth of the light source. Advances in technology have introduced devices with programmable settings that allow for precise control of these parameters. However, achieving uniformity in clinical practice requires the development and implementation of comprehensive guidelines that define the optimal parameters for different types of skin cancers and stages of disease progression. Collaborative efforts from researchers, clinicians, and regulatory authorities are required to create these standards. Moreover, continuous training and education of healthcare providers should be emphasized to ensure the correct application of these standards in clinical practice.

## 7. Conclusions

The status of skin cancer treatment varies depending on the type and stage of skin cancer. The three most common types of skin cancer, BCC, SCC, and melanoma, are treated with different approaches. For example, BCC (the most common type of skin cancer) is often localized and rarely spreads to other parts of the body. For this reason, treatment options for BCC include excision, Mohs surgery, cryosurgery, radiation therapy, and topical medications. On the other hand, SCC is typically localized but has a higher chance of spreading compared to BCC. In this case, the treatment options are similar to that of BCC, but in some cases, topical chemotherapy or immunotherapy creams may be used. Finally, melanoma is the most aggressive type of skin cancer and has a higher risk of spreading to other parts of the body. The treatment options depend on the stage and may include surgery, immunotherapy, targeted therapy, radiation therapy, and chemotherapy.

Difficulties faced in skin cancer treatment can include early detection challenges, the risk of metastasis, recurrence, treatment side effects, and limited access to healthcare in certain areas. Early detection of skin cancer can be challenging, especially in inconspicuous areas or when it resembles benign skin lesions. If skin cancer spreads to other parts of the body, it becomes more challenging to treat. Skin cancer can sometimes recur even after successful treatment, requiring further intervention, and some treatment modalities, such as surgery, radiation therapy, or systemic therapies, can have serious side effects. Moreover, the cosmetic outcomes of previous approaches are typically unsatisfactory, as substantial tissue often has to be removed or damaged. In this regard, PDT is a promising treatment option for skin cancer and pre-cancers, providing selectivity and effectiveness through photosensitizing agents and light while avoiding previous side effects (one of the main characteristics is the preservation of connective tissue). Although only a few PSs have been approved for skin cancer treatment, advanced techniques, such as artificial dPDT and metronomic PDT, offer potential benefits over conventional PDT.

To optimize the PDT process, maximizing the overlap volume integral of the diseased tissue, the molecular oxygen, the photosensitizer, and the illuminating light volumetric distributions is necessary. Appropriately modulating the light beam parameters can prevent oxygen depletion. An adequate signal pattern of the amplitude-modulated light beam can be used to recover potential oxygen depletions in the diseased cancer volume during the maximum intensity part of the illumination period.

Additionally, advanced light delivery devices, such as textile light diffusers, woven-based light-emitting fabrics, and plastics and adhesives, offer advantages over traditional light delivery methods, including improved flexibility, patient comfort, and precision in targeting tumor sites. As these technologies continue to develop, they may become increasingly important tools in the field of cancer and other disease treatments. However, further research is needed to refine treatment protocols, increase accessibility, and develop new light sources and PS for these approaches.

## Figures and Tables

**Table 1:** Approved PSs (not only for skin).

PS/ Drug Substance	λ (nm)	Time to PDT/ Clearance	Clinical/Preclinical Application	Side Effects	Localization	Primary Mechanism of Action	Refs
Photofrin^®^/Porfimer sodium	630	24–48 h/ 4–12 weeks [60]	**Approved** Esophageal cancer, lung cancer, microinvasive endobronchial cancer, gastric and papillary bladder, and cervical dysplasia and cancer	Mild to moderate erythema. Photosensitivity, mild constipation	Mitochondrial membrane and lysosome [61] Golgi apparatus [55]	Ischemic cell necrosis [55] Rapid illumination post infusion will favor vascular shutdown [62]	[55,56,58,59]
Foscan^®^/ Temoporfin (mTHPC)	652	48–96 h/ 24 h dark 1–7 days home [63]	**Preclinical testing:** Pancreas, breast **Approved:** Head and neck cancer.	Swelling, bleeding, ulceration, scarring Photosensitivity	Mitochondria [11] Endoplasmic reticulum (ER) [55]	Vascular damage and direct tumor cytotoxicity [55]	[11,60,61,62,63]
Ameluz^®^, Levulan^®^/5-ALA	410	1–3 h/ 48 h [64]	**Approved:** Actinic keratoses	Stinging, burning, itching, erythema	Mitochondria, cytosol, cytosolic membranes	Direct tumor cytotoxicity	[55,64,65,66]
Metvix^®^/Metvixia^®^ MAL	635	3 h/ 24 h	**Approved:** Actinic keratosis, basal cell carcinoma	Burning sensation, redness, scabbing	Mitochondria, cytosol, cytosolic membranes	Direct tumor cytotoxicity	[55,67]
Laserphyrin^®^/NPe6/Talaporfin	664	2–4 h/ 48 h dark 8–14 days home [65]	**Approved:** Early lung cancer **Clinical trials** Hepatocellular cancer, liver metastasis	Mild inflammatory Mild photodermatitis [66]	Lysosome, endosome	Vascular stasis and direct tumor cytotoxicity	[66,68,69]
Visudyne^®^/Verteporfin	690	15 min	**Approved:** Age-related macular degeneration		DNA fragmentation [11] Mitochondria membrane [67]		[11,70,71,72]
TOOKAD^®^/pheophorbides	753	15 min/ 24–48 h [68]	**Clinical trial:** prostate cancer	-	Vascular occlusion [69]	Vascular damage [55]	[55,73,74]

**Table 2:** The benefits and drawbacks of utilizing coherent light sources (such as lasers) versus non-coherent sources (like LED and lamps) for superficial PDT [78].

Light Source	Advantages	Disadvantages
**Laser** (coherent)	<0.1 nm spectral bandwidth High power Efficient coupling to optical fibres Uniform irradiance can be easily achieved Adaptive emission (VCSEL, Edge-emitting laser) Faster modulation than LEDs	Only for small areas
**LED** (non-coherent)	Low cost Small Adaptive emission (SLED, ELED) Used for whole-body or point treatment LEDs can fit down biopsy channels, permitting deep-seated PDT	5–10 nm spectral bandwidth Large beam divergence
**Lamp** (non-coherent)	Low cost Simple design Wide illumination field Multi-photon irradiance	UV and NIR radiation (optical filtering is needed) Large beam divergence High coupling losses with light guides

## Data Availability

Data will be available upon reasonable request.

## References

[1] American Cancer Society Key Statistics for Basal and Squamous Cell Skin Cancers. https://www.cancer.org/cancer/basal-and-squamous-cell-skin-cancer/about/key-statistics.html.

[2] del Puerto A., Hernández-Rodriguez J.C., Sendín-Martín M., Ortiz-Alvarez J., Conejo-Mir Sánchez J., Pereyra-Rodriguez J.J. (2023). Skin Cancer Mortality in Spain: Adjusted Mortality Rates by Province and Related Risk Factors. Int. J. Dermatol..

[3] Tejera-Vaquerizo A., Descalzo-Gallego M.A., Otero-Rivas M.M., Posada-García C., Rodríguez-Pazos L., Pastushenko I., Marcos-Gragera R., García-Doval I. (2016). Incidencia y Mortalidad Del Cáncer Cutáneo En España: Revisión Sistemática y Metaanálisis. Actas Dermosifiliogr..

[4] Ackroyd R., Kelty C., Brown N., Reed M. (2001). The History of Photodetection and Photodynamic Therapy. Photochem. Photobiol..

[5] Kessel D. (2019). Photodynamic Therapy: A Brief History. J. Clin. Med..

[6] Keyal U., Bhatta A.K., Zhang G., Wang X.L. (2019). Present and Future Perspectives of Photodynamic Therapy for Cutaneous Squamous Cell Carcinoma. J. Am. Acad. Dermatol..

[7] Zhou Z., Song J., Nie L., Chen X. (2016). Reactive Oxygen Species Generating Systems Meeting Challenges of Photodynamic Cancer Therapy. Chem. Soc. Rev..

[8] Castano A.P., Demidova T.N., Hamblin M.R. (2005). Mechanisms in Photodynamic Therapy: Part Three—Photosensitizer Pharmacokinetics, Biodistribution, Tumor Localization and Modes of Tumor Destruction. Photodiagnosis Photodyn. Ther..

[9] Nowis D., Makowski M., Stokłosa T., Legat M., Issat T., Gołab J. (2005). Direct Tumor Damage Mechanisms of Photodynamic Therapy. Acta Biochim. Pol..

[10] Mroz P., Yaroslavsky A., Kharkwal G.B., Hamblin M.R. (2011). Cell Death Pathways in Photodynamic Therapy of Cancer. Cancers.

[11] Castano A.P., Demidova T.N., Hamblin M.R. (2005). Mechanisms in Photodynamic Therapy: Part Two—Cellular Signaling, Cell Metabolism and Modes of Cell Death. Photodiagnosis Photodyn. Ther..

[12] Szeimies R.-M., Radny P., Sebastian M., Borrosch F., Dirschka T., Krähn-Senftleben G., Reich K., Pabst G., Voss D., Foguet M. (2010). Photodynamic Therapy with BF-200 ALA for the Treatment of Actinic Keratosis: Results of a Prospective, Randomized, Double-Blind, Placebo-Controlled Phase III Study. Br. J. Dermatol..

[13] Morton C.A. (2007). Methyl Aminolevulinate: Actinic Keratoses and Bowen’s Disease. Dermatol. Clin..

[14] Allison R.R., Sibata C.H. (2010). Photodynamic Therapy: Mechanism of Action and Role in the Treatment of Skin Disease. G. Ital. Dermatol. Venereol..

[15] Allison R.R. (2014). Photodynamic Therapy: Oncologic Horizons. Futur. Oncol..

[16] Holmes C., Foley P., Freeman M., Chong A.H. (2007). Solar Keratosis: Epidemiology, Pathogenesis, Presentation and Treatment. Australas. J. Dermatol..

[17] Stockfleth E., Ferrandiz C., Grob J.J., Leigh I., Pehamberger H., Kerl H. (2008). Development of a Treatment Algorithm for Actinic Keratoses: A European Consensus. Eur. J. Dermatol..

[18] Wan M.T., Lin J.Y. (2014). Current Evidence and Applications of Photodynamic Therapy in Dermatology. Clin. Cosmet. Investig. Dermatol..

[19] Fayter D., Corbett M., Heirs M., Fox D., Eastwood A. (2010). A Systematic Review of Photodynamic Therapy in the Treatment of Precancerous Skin Conditions, Barrett’s Oesophagus and Cancers of the Biliary Tract, Brain, Head and Neck, Lung, Oesophagus and Skin. Health Technol. Assess..

[20] Zhao B., He Y.-Y. (2010). Recent Advances in the Prevention and Treatment of Skin Cancer Using Photodynamic Therapy. Expert Rev. Anticancer Ther..

[21] Basset-Seguin N., Ibbotson S.H., Emtestam L., Tarstedt M., Morton C., Maroti M., Calzavara-Pinton P., Varma S., Roelandts R., Wolf P. (2008). Topical Methyl Aminolaevulinate Photodynamic Therapy versus Cryotherapy for Superficial Basal Cell Carcinoma: A 5 Year Randomized Trial. Eur. J. Dermatol..

[22] Wang B.C., Fu C., Qin L., Zeng X.Y., Liu Q. (2020). Photodynamic Therapy with Methyl-5-Aminolevulinate for Basal Cell Carcinoma: A Systematic Review and Meta-Analysis. Photodiagnosis Photodyn. Ther..

[23] Horlings R.K., Terra J.B., Witjes M.J.H. (2015). MTHPC Mediated, Systemic Photodynamic Therapy (PDT) for Nonmelanoma Skin Cancers: Case and Literature Review. Lasers Surg. Med..

[24] Lehmann P. (2007). Methyl Aminolaevulinate-Photodynamic Therapy: A Review of Clinical Trials in the Treatment of Actinic Keratoses and Nonmelanoma Skin Cancer. Br. J. Dermatol..

[25] Morton C., Horn M., Leman J., Tack B., Bedane C., Tjioe M., Ibbotson S., Khemis A., Wolf P. (2006). Comparison of Topical Methyl Aminolevulinate Photodynamic Therapy with Cryotherapy or Fluorouracil for Treatment of Squamous Cell Carcinoma In Situ: Results of a Multicenter Randomized Trial. Arch. Dermatol..

[26] Truchuelo M., Fernández-Guarino M., Fleta B., Alcántara J., Jaén P. (2012). Effectiveness of Photodynamic Therapy in Bowen’s Disease: An Observational and Descriptive Study in 51 Lesions. J. Eur. Acad. Dermatol. Venereol..

[27] Calzavara-Pinton P.G., Venturini M., Sala R., Capezzera R., Parrinello G., Specchia C., Zane C. (2008). Methylaminolaevulinate-Based Photodynamic Therapy of Bowen’s Disease and Squamous Cell Carcinoma. Br. J. Dermatol..

[28] Cavicchini S., Serini S.M., Fiorani R., Girgenti V., Ghislanzoni M., Sala F. (2011). Long-Term Follow-up of Metil Aminolevulinate (MAL)-PDT in Difficult-to-Treat Cutaneous Bowen’s Disease. Int. J. Dermatol..

[29] Wang X., Shen S., Ning C., Xu M., Yan X. (2015). A Sparse Representation-Based Method for Infrared Dim Target Detection under Sea–Sky Background. Infrared Phys. Technol..

[30] Wang X., Shi L., Tu Q., Wang H., Zhang H., Wang P., Zhang L., Huang Z., Zhao F., Luan H. (2015). Treating Cutaneous Squamous Cell Carcinoma Using 5-Aminolevulinic Acid Polylactic-Co-Glycolic Acid Nanoparticle-Mediated Photodynamic Therapy in a Mouse Model. Int. J. Nanomed..

[31] Lucena S.R., Salazar N., Gracia-Cazaña T., Zamarrón A., González S., Juarranz Á., Gilaberte Y. (2015). Combined Treatments with Photodynamic Therapy for Non-Melanoma Skin Cancer. Int. J. Mol. Sci..

[32] Erkiert-Polguj A., Halbina A., Polak-Pacholczyk I., Rotsztejn H. (2016). Light-Emitting Diodes in Photodynamic Therapy in Non-Melanoma Skin Cancers—Own Observations and Literature Review. J. Cosmet. Laser Ther..

[33] Marmur E.S., Schmults C.D., Goldberg D.J. (2004). A Review of Laser and Photodynamic Therapy for the Treatment of Nonmelanoma Skin Cancer. Dermatol. Surg..

[34] Beiki D., Eggleston I.M., Pourzand C. (2022). Daylight-PDT: Everything under the Sun. Biochem. Soc. Trans..

[35] Lee C.-N., Hsu R., Chen H., Wong T.-W. (2020). Daylight Photodynamic Therapy: An Update. Molecules.

[36] O’Mahoney P., Khazova M., Eadie E., Ibbotson S. (2019). Measuring Daylight: A Review of Dosimetry in Daylight Photodynamic Therapy. Pharmaceuticals.

[37] Tylcz J.B., Vicentini C., Mordon S. (2016). Light emitting textiles for a photodynamic therapy. Smart Textiles and Their Applications.

[38] Mordon S., Cochrane C., Tylcz J.B., Betrouni N., Mortier L., Koncar V. (2015). Light Emitting Fabric Technologies for Photodynamic Therapy. Photodiagnosis Photodyn. Ther..

[39] Mordon S., Thécua E., Ziane L., Lecomte F., Deleporte P., Baert G., Vignion-Dewalle A. (2020). Light Emitting Fabrics for Photodynamic Therapy: Technology, Experimental and Clinical Applications. Transl. Biophotonics.

[40] Wen X., Li Y., Hamblin M.R. (2017). Photodynamic Therapy in Dermatology beyond Non-Melanoma Cancer: An Update. Photodiagnosis Photodyn. Ther..

[41] Tampa M., Sarbu M.I., Matei C., Mitran C.I., Mitran M.I., Caruntu C., Constantin C., Neagu M., Georgescu S.R. (2019). Photodynamic Therapy: A Hot Topic in Dermato-Oncology (Review). Oncol. Lett..

[42] Yu X., Zheng H., Chan M.T.V., Wu W.K.K. (2018). Immune Consequences Induced by Photodynamic Therapy in Non-Melanoma Skin Cancers: A Review. Environ. Sci. Pollut. Res..

[43] Algorri J.F., Ochoa M., Roldán-Varona P., Rodríguez-Cobo L., López-Higuera J.M. (2021). Photodynamic Therapy: A Compendium of Latest Reviews. Cancers.

[44] Li W.-P., Yen C.-J., Wu B.-S., Wong T.-W. (2021). Recent Advances in Photodynamic Therapy for Deep-Seated Tumors with the Aid of Nanomedicine. Biomedicines.

[45] Michael R. (2017). Hamblin Imaging in Photodynamic Therapy.

[46] Valeur B., Berberan-Santos M.N. (2012). Molecular Fluorescence: Principles and Applications.

[47] Plaetzer K., Krammer B., Berlanda J., Berr F., Kiesslich T. (2009). Photophysics and Photochemistry of Photodynamic Therapy: Fundamental Aspects. Lasers Med. Sci..

[48] Broekgaarden M., Weijer R., van Gulik T.M., Hamblin M.R., Heger M. (2015). Tumor Cell Survival Pathways Activated by Photodynamic Therapy: A Molecular Basis for Pharmacological Inhibition Strategies. Cancer Metastasis Rev..

[49] Robertson C.A., Evans D.H., Abrahamse H. (2009). Photodynamic Therapy (PDT): A Short Review on Cellular Mechanisms and Cancer Research Applications for PDT. J. Photochem. Photobiol. B Biol..

[50] Yoo J.O., Ha K.S. (2012). New Insights into the Mechanisms for Photodynamic Therapy-Induced Cancer Cell Death. International Review of Cell and Molecular Biology.

[51] Kushibiki T., Hirasawa T., Okawa S., Ishihara M. (2013). Responses of Cancer Cells Induced by Photodynamic Therapy. J. Healthc. Eng..

[52] Allison R.R., Moghissi K. (2013). Photodynamic Therapy (PDT): PDT Mechanisms. Clin. Endosc..

[53] Dahle J., Kaalhus O., Moan J., Steen H.B. (1997). Cooperative Effects of Photodynamic Treatment of Cells in Microcolonies. Proc. Natl. Acad. Sci. USA.

[54] Henderson B.W., Busch T.M., Snyder J.W. (2006). Fluence Rate as a Modulator of PDT Mechanisms. Lasers Surg. Med..

[55] O’Connor A.E., Gallagher W.M., Byrne A.T. (2009). Porphyrin and Nonporphyrin Photosensitizers in Oncology: Preclinical and Clinical Advances in Photodynamic Therapy. Photochem. Photobiol..

[56] Mfouo-Tynga I.S., Dias L.D., Inada N.M., Kurachi C. (2021). Features of Third Generation Photosensitizers Used in Anticancer Photodynamic Therapy: Review. Photodiagnosis Photodyn. Ther..

[57] Gomez S., Tsung A., Hu Z. (2020). Current Targets and Bioconjugation Strategies in Photodynamic Diagnosis and Therapy of Cancer. Molecules.

[58] Mesquita M.Q., Dias C.J., Gamelas S., Fardilha M., Neves M.G.P.M.S., Faustino M.A.F. (2018). An Insight on the Role of Photosensitizer Nanocarriers for Photodynamic Therapy. An. Acad. Bras. Cienc..

[59] Park W., Cho S., Han J., Shin H., Na K., Lee B., Kim D.-H. (2018). Advanced Smart-Photosensitizers for More Effective Cancer Treatment. Biomater. Sci..

[60] Khan S.A., Dougherty T.J., Mang T.S. (1993). An Evaluation of Photodynamic Therapy in the Management of Cutaneous Metastases of Breast Cancer. Eur. J. Cancer.

[61] Kessel D. (2016). Photodynamic Therapy: Promotion of Efficacy by a Sequential Protocol. J. Porphyr. Phthalocyanines.

[62] Allison R.R., Sibata C.H. (2010). Oncologic Photodynamic Therapy Photosensitizers: A Clinical Review. Photodiagnosis Photodyn. Ther..

[63] Biogen Inc Annex I Summary of Product Characteristics Tecfidera. https://ec.europa.eu/health/documents/community-register/2015/20151216133545/anx_133545_en.pdf.

[64] Ericson M.B., Wennberg A.M., Larkö O. (2008). Review of Photodynamic Therapy in Actinic Keratosis and Basal Cell Carcinoma. Ther. Clin. Risk Manag..

[65] Nakamura T., Oinuma T. (2014). Usefulness of Photodynamic Diagnosis and Therapy Using Talaporfin Sodium for an Advanced-Aged Patient with Inoperable Gastric Cancer (a Secondary Publication). Laser Ther..

[66] Nanashima A., Abo T., Nonaka T., Nonaka Y., Morisaki T., Uehara R., Ohnita K.E.N., Fukuda D., Murakami G., Tou K. (2012). Photodynamic Therapy Using Talaporfin Sodium (Laserphyrin^®^) for Bile Duct Carcinoma: A Preliminary Clinical Trial. Anticancer Res..

[67] Mahalingam S.M., Ordaz J.D., Low P.S. (2018). Targeting of a Photosensitizer to the Mitochondrion Enhances the Potency of Photodynamic Therapy. ACS Omega.

[68] Azzouzi A.R., Barret E., Moore C.M., Villers A., Allen C., Scherz A., Muir G., De Wildt M., Barber N.J., Lebdai S. (2013). TOOKAD^®^ Soluble Vascular-Targeted Photodynamic (VTP) Therapy: Determination of Optimal Treatment Conditions and Assessment of Effects in Patients with Localised Prostate Cancer. BJU Int..

[69] Banerjee S.M.M., MacRobert A.J.J., Mosse C.A.A., Periera B., Bown S.G.G., Keshtgar M.R.S.R.S. (2017). Photodynamic Therapy: Inception to Application in Breast Cancer.

[70] Kim M.M., Darafsheh A. (2020). Light Sources and Dosimetry Techniques for Photodynamic Therapy. Photochem. Photobiol..

[71] Brancaleon L., Moseley H. (2002). Laser and Non-Laser Light Sources for Photodynamic Therapy. Lasers Med. Sci..

[72] Mang T.S. (2004). Lasers and Light Sources for PDT: Past, Present and Future. Photodiagnosis Photodyn. Ther..

[73] Katarina S., Bendsoe N., Axelsson J., Andersson-Engels S., Svanberg S. (2010). Photodynamic Therapy: Superficial and Interstitial Illumination. J. Biomed. Opt..

[74] Liu H., Daly L., Rudd G., Khan A.P., Mallidi S., Liu Y., Cuckov F., Hasan T., Celli J.P. (2019). Development and Evaluation of a Low-Cost, Portable, LED-Based Device for PDT Treatment of Early-Stage Oral Cancer in Resource-Limited Settings. Lasers Surg. Med..

[75] Vu H., Kieu N.M., Gam D.T., Shin S., Tien T.Q., Vu N.H. (2020). Design and Evaluation of Uniform LED Illumination Based on Double Linear Fresnel Lenses. Appl. Sci..

[76] Boucher D., Longo L. (2011). The Tools of PDT: Light Sources and Devices. Can They Help in Getting Better Therapeutic Results?.

[77] Nagata J.Y., Hioka N., Kimura E., Batistela V.R., Terada R.S.S., Graciano A.X., Baesso M.L., Hayacibara M.F. (2012). Antibacterial Photodynamic Therapy for Dental Caries: Evaluation of the Photosensitizers Used and Light Source Properties. Photodiagnosis Photodyn. Ther..

[78] Algorri J.F., Ochoa M., Roldán-Varona P., Rodríguez-Cobo L., López-Higuera J.M. (2021). Light Technology for Efficient and Effective Photodynamic Therapy: A Critical Review. Cancers.

[79] Woodburn K.W., Young S.W., Qing F., Miles D.R., Thiemann P.A., Dougherty T.J. (1997). Light Emitting Diode versus Laser Irradiation Phototherapy with Lutetium Texaphyrin (PCI-0123). Proceedings of the Optical Methods for Tumor Treatment and Detection: Mechanisms and Techniques in Photodynamic Therapy VI.

[80] Yu C.H., Lin H.P., Chen H.M., Yang H., Wang Y.P., Chiang C.P. (2009). Comparison of Clinical Outcomes of Oral Erythroleukoplakia Treated with Photodynamic Therapy Using Either Light-Emitting Diode or Laser Light. Lasers Surg. Med..

[81] De Jode M.L., McGilligan J.A., Dilkes M.G., Cameron I., Hart P.B., Grahn M.F. (1997). A Comparison of Novel Light Sources for Photodynamic Therapy. Proceedings of the Lasers in Medical Science.

[82] Ahluwalia J., Avram M.M., Ortiz A.E. (2020). The Evolving Story of Laser Therapeutics for Basal Cell Carcinoma. Dermatologic Surg..

[83] Vicentini C., Vignion-Dewalle A.S., Thecua E., Lecomte F., Maire C., Deleporte P., Béhal H., Kerob D., Duhamel A., Mordon S. (2019). Photodynamic Therapy for Actinic Keratosis of the Forehead and Scalp: A Randomized, Controlled, Phase II Clinical Study Evaluating the Noninferiority of a New Protocol Involving Irradiation with a Light-emitting, Fabric-based Device (the Flexitheralight P. Br. J. Dermatol..

[84] Ratycz M.C., Lender J.A., Gottwald L.D. (2019). Multiple Dorsal Hand Actinic Keratoses and Squamous Cell Carcinomas: A Unique Presentation Following Extensive UV Nail Lamp Use. Case Rep. Dermatol..

[85] Maire C., Vignion-Dewalle A.S., Cartier H., Mordon S. (2020). Artificial White Light Photodynamic Therapy for Actinic Keratosis: A Study of 38 Patients in Private Office Practice. J. Eur. Acad. Dermatol. Venereol..

[86] Zeitouni N.C., Oseroff A.R., Shieh S. (2003). Photodynamic Therapy for Nonmelanoma Skin Cancers: Current Review and Update. Proceedings of the Molecular Immunology.

[87] Wiegell S.R., Heydenreich J., Fabricius S., Wulf H.C. (2011). Continuous Ultra-Low-Intensity Artificial Daylight Is Not as Effective as Red LED Light in Photodynamic Therapy of Multiple Actinic Keratoses. Photodermatol. Photoimmunol. Photomed..

[88] Choudhary S., Tang J., Elsaie M.L., Nouri K. (2011). Lasers in the Treatment of Nonmelanoma Skin Cancer. Dermatol. Surg..

[89] Farberg A.S., Marson J.W., Soleymani T. (2023). Advances in Photodynamic Therapy for the Treatment of Actinic Keratosis and Nonmelanoma Skin Cancer: A Narrative Review. Dermatol. Ther..

[90] Steeb T., Schlager J.G., Kohl C., Ruzicka T., Heppt M.V., Berking C. (2019). Laser-Assisted Photodynamic Therapy for Actinic Keratosis: A Systematic Review and Meta-Analysis. J. Am. Acad. Dermatol..

[91] McLellan L.J., O’Mahoney P., Khazova M., Higlett M., Ibbotson S.H., Eadie E. (2019). Ultraviolet Radiation Exposure during Daylight Photodynamic Therapy. Photodiagnosis Photodyn. Ther..

[92] Fitzmaurice S., Eisen D.B. (2016). Daylight Photodynamic Therapy: What Is Known and What Is Yet to Be Determined. Dermatologic Surg..

[93] Lesar A., Ferguson J., Moseley H. (2011). An Investigation of the Fluorescence Induced by Topical Application of 5-Aminolaevulinic Acid and Methyl Aminolaevulinate at Different Body Sites on Normal Human Skin. Photodiagnosis Photodyn. Ther..

[94] Morton C.A., Szeimies R.-M., Braathen L.R. (2021). Review of the European Society for Photodynamic Therapy (Euro-PDT) Annual Congress 2020. Eur. J. Dermatol..

[95] Wiegell S.R., Haedersdal M., Philipsen P.A., Eriksen P., Enk C.D., Wulf H.C. (2008). Continuous Activation of PpIX by Daylight Is as Effective as and Less Painful than Conventional Photodynamic Therapy for Actinic Keratoses; a Randomized, Controlled, Single-Blinded Study. Br. J. Dermatol..

[96] Wiegell S.R., Haedersdal M., Eriksen P., Wulf H.C. (2009). Photodynamic Therapy of Actinic Keratoses with 8% and 16% Methyl Aminolaevulinate and Home-Based Daylight Exposure: A Double-Blinded Randomized Clinical Trial. Br. J. Dermatol..

[97] Wiegell S.R., Fabricius S., Stender I.M., Berne B., Kroon S., Andersen B.L., Mørk C., Sandberg C., Jemec G.B.E., Mogensen M. (2011). A Randomized, Multicentre Study of Directed Daylight Exposure Times of 1½ vs. 2½ h in Daylight-Mediated Photodynamic Therapy with Methyl Aminolaevulinate in Patients with Multiple Thin Actinic Keratoses of the Face and Scalp. Br. J. Dermatol..

[98] Wiegell S.R., Fabricius S., Gniadecka M., Stender I.M., Berne B., Kroon S., Andersen B.L., Mørk C., Sandberg C., Ibler K.S. (2012). Daylight-Mediated Photodynamic Therapy of Moderate to Thick Actinic Keratoses of the Face and Scalp: A Randomized Multicentre Study. Br. J. Dermatol..

[99] Rubel D.M., Spelman L., Murrell D.F., See J.-A., Hewitt D., Foley P., Bosc C., Kerob D., Kerrouche N., Wulf H.C. (2014). Daylight Photodynamic Therapy with Methyl Aminolevulinate Cream as a Convenient, Similarly Effective, Nearly Painless Alternative to Conventional Photodynamic Therapy in Actinic Keratosis Treatment: A Randomized Controlled Trial. Br. J. Dermatol..

[100] Zeitouni N.C., Paquette A.D., Housel J.P., Shi Y., Wilding G.E., Foster T.H., Henderson B.W. (2013). A Retrospective Review of Pain Control by a Two-Step Irradiance Schedule during Topical ALA-Photodynamic Therapy of Non-Melanoma Skin Cancer. Lasers Surg. Med..

[101] Anand S., Yasinchak A., Govande M., Shakya S., Maytin E. (2019). V Painless versus Conventional Photodynamic Therapy for Treatment of Actinic Keratosis: Comparison of Cell Death and Immune Response in a Murine Model. Proc. SPIE Int. Soc. Opt. Eng..

[102] Salido-Vallejo R., Jiménez-Nájar F., Garnacho-Sucedo G., Vélez A. (2020). Combined Daylight and Conventional Photodynamic Therapy with 5-Aminolaevulinic Acid Nanoemulsion (BF-200 ALA) for Actinic Keratosis of the Face and Scalp: A New and Efficient Approach. Arch. Dermatol. Res..

[103] Marra K., LaRochelle E.P., Chapman M.S., Hoopes P.J., Lukovits K., Maytin E.V., Hasan T., Pogue B.W. (2018). Comparison of Blue and White Lamp Light with Sunlight for Daylight-Mediated, 5-ALA Photodynamic Therapy, in Vivo. Photochem. Photobiol..

[104] Lerche C.M., Heerfordt I.M., Heydenreich J., Wulf H.C. (2016). Alternatives to Outdoor Daylight Illumination for Photodynamic Therapy—Use of Greenhouses and Artificial Light Sources. Int. J. Mol. Sci..

[105] O’Mahoney P., Haigh N., Wood K., Brown C.T.A., Ibbotson S., Eadie E. (2018). A Novel Light Source with Tuneable Uniformity of Light Distribution for Artificial Daylight Photodynamic Therapy. Photodiagnosis Photodyn. Ther..

[106] Bisland S.K., Lilge L., Lin A., Rusnov R., Wilson B.C. (2004). Metronomic Photodynamic Therapy as a New Paradigm for Photodynamic Therapy: Rationale and Preclinical Evaluation of Technical Feasibility for Treating Malignant Brain Tumors. Photochem. Photobiol..

[107] Wilson B.C., Bisland S.K., Bogaards A., Lin A., Moriyama E.H., Zhang K., Lilge L.D. Metronomic Photodynamic Therapy (*m*PDT): Concepts and Technical Feasibility in Brain Tumor. Proceedings of the Optical Methods for Tumor Treatment and Detection: Mechanisms and Techniques in Photodynamic Therapy XII.

[108] Anand S., Govande M., Yasinchak A., Heusinkveld L., Shakya S., Maytin E. V Metronomic PDT Induces Innate and Adaptive Immune Responses in Murine Models of Skin Cancer and Pre-Cancer. Proceedings of the Optical Methods for Tumor Treatment and Detection: Mechanisms and Techniques in Photodynamic Therapy XXIX.

[109] Dai J., Wei S., Xu J., Xue H., Chen Z., Wu M., Chen W., Lou X., Xia F., Wang S. (2023). Microneedle Device Delivering Aggregation-Induced Emission Photosensitizers for Enhanced Metronomic Photodynamic Therapy of Cancer. ACS Appl. Mater. Interfaces.

[110] Singh G., Alqawi O., Espiritu M., Gomer C.J. (2010). Metronomic PDT and Cell Death Pathways BT—Photodynamic Therapy: Methods and Protocols.

[111] Yanovsky R.L., Bartenstein D.W., Rogers G.S., Isakoff S.J., Chen S.T. (2019). Photodynamic Therapy for Solid Tumors: A Review of the Literature. Photodermatol. Photoimmunol. Photomed..

[112] Messmann H., Mlkvy P., Buonaccorsi G., Davies C.L., MacRobert A.J., Bown S.G. (1995). Enhancement of Photodynamic Therapy with 5-Aminolaevulinic Acid-Induced Porphyrin Photosensitisation in Normal Rat Colon by Threshold and Light Fractionation Studies. Br. J. Cancer.

[113] Müller S., Walt H., Dobler-Girdziunaite D., Fiedler D., Haller U. (1998). Enhanced Photodynamic Effects Using Fractionated Laser Light. J. Photochem. Photobiol. B Biol..

[114] de Haas E.R.M., Kruijt B., Sterenborg H.J.C.M., Martino Neumann H.A., Robinson D.J. (2006). Fractionated Illumination Significantly Improves the Response of Superficial Basal Cell Carcinoma to Aminolevulinic Acid Photodynamic Therapy. J. Investig. Dermatol..

[115] de Haas E.R.M., Sterenborg H.J.C.M., Neumann H.A.M., Robinson D.J. (2007). Response of Bowen Disease to ALA-PDT Using a Single and a 2-Fold Illumination Scheme. Arch. Dermatol..

[116] de Vijlder H.C., Sterenborg H.J.C.M., Neumann H.A.M., Robinson D.J., de Haas E.R.M. (2012). Light Fractionation Significantly Improves the Response of Superficial Basal Cell Carcinoma to Aminolaevulinic Acid Photodynamic Therapy: Five-Year Follow-up of a Randomized, Prospective Trial. Acta Derm. Venereol..

[117] Sotiriou E., Apalla Z., Chovarda E., Goussi C., Trigoni A., Ioannides D. (2012). Single vs. Fractionated Photodynamic Therapy for Face and Scalp Actinic Keratoses: A Randomized, Intraindividual Comparison Trial with 12-Month Follow-Up. J. Eur. Acad. Dermatol. Venereol..

[118] de Haas E.R.M., de Vijlder H.C., Sterenborg H.J.C.M., Neumann H.A.M., Robinson D.J. (2008). Fractionated Aminolevulinic Acid-Photodynamic Therapy Provides Additional Evidence for the Use of PDT for Non-Melanoma Skin Cancer. J. Eur. Acad. Dermatol. Venereol..

[119] Mosterd K., Thissen M.R.T.M., Nelemans P., Kelleners-Smeets N.W.J., Janssen R.L.L.T., Broekhof K.G.M.E., Neumann H.A.M., Steijlen P.M., Kuijpers D.I.M. (2008). Fractionated 5-Aminolaevulinic Acid-Photodynamic Therapy vs. Surgical Excision in the Treatment of Nodular Basal Cell Carcinoma: Results of a Randomized Controlled Trial. Br. J. Dermatol..

[120] Kessels J.P.H.M., Kreukels H., Nelemans P.J., Roozeboom M.H., van Pelt H., Mosterd K., de Haas E.R.M., Kelleners-Smeets N.W.J. (2018). Treatment of Superficial Basal Cell Carcinoma by Topical Photodynamic Therapy with Fractionated 5-Aminolaevulinic Acid 20% vs. Two-Stage Topical Methyl Aminolaevulinate: Results of a Randomized Controlled Trial. Br. J. Dermatol..

[121] Ko D.-Y., Jeon S.-Y., Kim K.-H., Song K.-H. (2014). Fractional Erbium: YAG Laser-Assisted Photodynamic Therapy for Facial Actinic Keratoses: A Randomized, Comparative, Prospective Study. J. Eur. Acad. Dermatol. Venereol..

[122] Bhawalkar J.D., Kumar N.D., Zhao C.-F., Prasad P.N. (1997). Two-Photon Photodynamic Therapy. J. Clin. Laser Med. Surg..

[123] Khurana M., Collins H.A., Karotki A., Anderson H.L., Cramb D.T., Wilson B.C. (2007). Quantitative In Vitro Demonstration of Two-Photon Photodynamic Therapy Using Photofrin^®^ and Visudyne^®^. Photochem. Photobiol..

[124] Heinemann F., Karges J., Gasser G. (2017). Critical Overview of the Use of Ru(II) Polypyridyl Complexes as Photosensitizers in One-Photon and Two-Photon Photodynamic Therapy. Acc. Chem. Res..

[125] Ogawa K., Kobuke Y. (2008). Recent Advances in Two-Photon Photodynamic Therapy. Anticancer. Agents Med. Chem..

[126] Sun Z., Zhang L.-P., Wu F., Zhao Y. (2017). Photosensitizers for Two-Photon Excited Photodynamic Therapy. Adv. Funct. Mater..

[127] Xu L., Zhang J., Yin L., Long X., Zhang W., Zhang Q. (2020). Recent Progress in Efficient Organic Two-Photon Dyes for Fluorescence Imaging and Photodynamic Therapy. J. Mater. Chem. C.

[128] Shen Y., Shuhendler A.J., Ye D., Xu J.J., Chen H.Y. (2016). Two-Photon Excitation Nanoparticles for Photodynamic Therapy. Chem. Soc. Rev..

[129] Rendon A., Weersink R., Lilge L. (2006). Towards Conformal Light Delivery Using Tailored Cylindrical Diffusers: Attainable Light Dose Distributions. Phys. Med. Biol..

[130] Endruweit A., Long A.C., Johnson M.S. (2007). Textile Composites with Integrated Optical Fibres: Quantification of the Influence of Single and Multiple Fibre Bends on the Light Transmission Using a Monte Carlo Ray-Tracing Method. Smart Mater. Struct..

[131] Selm B., Rothmaier M., Camenzind M., Khan T.N., Walt H. (2007). Novel Flexible Light Diffuser and Irradiation Properties for Photodynamic Therapy. J. Biomed. Opt..

[132] Cochrane C., Mordon S.R., Lesage J.C., Koncar V. (2013). New Design of Textile Light Diffusers for Photodynamic Therapy. Mater. Sci. Eng. C.

[133] Vignion-Dewalle A.-S., Abi Rached H., Thecua E., Lecomte F., Deleporte P., Béhal H., Hommel T., Duhamel A., Szeimies R.-M., Mortier L. (2019). A New Light-Emitting, Fabric-Based Device for Photodynamic Therapy of Actinic Keratosis: Protocol for a Randomized, Controlled, Multicenter, Intra-Individual, Phase II Noninferiority Study (the Phosistos Study). JMIR Res. Protoc..

[134] Lecomte F., Thecua E., Ziane L., Deleporte P., Duhamel A., Maire C., Staumont-Salle D., Mordon S., Mortier L. (2019). Photodynamic Therapy Using a New Painless Light-Emitting Fabrics Device in the Treatment of Extramammary Paget Disease of the Vulva (the PAGETEX Study): Protocol for an Interventional Efficacy and Safety Trial. JMIR Res. Protoc..

[135] Hu Y., Wang K., Zhu T.C. A Light Blanket for Intraoperative Photodynamic Therapy. Proceedings of the Photodynamic Therapy: Back to the Future.

[136] Hu Y., Wang K., Zhu T.C., Kessel D.H. Pre-Clinic Study of Uniformity of Light Blanket for Intraoperative Photodynamic Therapy. Proceedings of the Optical Methods for Tumor Treatment and Detection: Mechanisms and Techniques in Photodynamic Therapy XIX.

[137] Liang X., Kundu P., Finlay J., Goodwin M., Zhu T.C. Maximizing Fluence Rate and Field Uniformity of Light Blanket for Intraoperative PDT. Proceedings of the Optical Methods for Tumor Treatment and Detection: Mechanisms and Techniques in Photodynamic Therapy XXI.

[138] Masuda H., Kimura M., Nishioka A., Kato H., Morita A. (2019). Dual Wavelength 5-Aminolevulinic Acid Photodynamic Therapy Using a Novel Flexible Light-Emitting Diode Unit. J. Dermatol. Sci..

[139] Yamagishi K., Kirino I., Takahashi I., Amano H., Takeoka S., Morimoto Y., Fujie T. (2019). Tissue-Adhesive Wirelessly Powered Optoelectronic Device for Metronomic Photodynamic Cancer Therapy. Nat. Biomed. Eng..

[140] Chen H., Yeh T.-H., He J., Zhang C., Abbel R., Hamblin M.R., Huang Y., Lanzafame R.J., Stadler I., Celli J. (2018). Flexible Quantum Dot Light-Emitting Devices for Targeted Photomedical Applications. J. Soc. Inf. Disp..

[141] Chen H., He J., Lanzafame R., Stadler I., El Hamidi H., Liu H., Celli J., Hamblin M.R., Huang Y., Oakley E. (2017). Quantum Dot Light Emitting Devices for Photomedical Applications. J. Soc. Inf. Disp..

[142] Jeon Y., Noh I., Seo Y.C., Han J.H., Park Y., Cho E.H., Choi K.C. (2020). Parallel-Stacked Flexible Organic Light-Emitting Diodes for Wearable Photodynamic Therapeutics and Color-Tunable Optoelectronics. ACS Nano.

[143] Guo H.-W., Lin L.-T., Chen P.-H., Ho M.-H., Huang W.-T., Lee Y.-J., Chiou S.-H., Hsieh Y.-S., Dong C.-Y., Wang H.-W. (2015). Low-Fluence Rate, Long Duration Photodynamic Therapy in Glioma Mouse Model Using Organic Light Emitting Diode (OLED). Photodiagnosis Photodyn. Ther..

[144] Attili S.K., Lesar A., McNeill A., Camacho-Lopez M., Moseley H., Ibbotson S., Samuel I.D.W., Ferguson J. (2009). An Open Pilot Study of Ambulatory Photodynamic Therapy Using a Wearable Low-Irradiance Organic Light-Emitting Diode Light Source in the Treatment of Nonmelanoma Skin Cancer. Br. J. Dermatol..

